# Development of new bilingual oral health behavior social support (OHBSS) scales in English and Spanish

**DOI:** 10.1371/journal.pone.0317133

**Published:** 2025-03-11

**Authors:** Tracy L. Finlayson, Vanessa L. Malcarne, Guadalupe X. Ayala, Melody K. Schiaffino, Kristin S. Hoeft, Cristian Garcia-Alcaraz, Mark Ryder, Stuart A. Gansky, Lourdes S. Martinez, Mingan Yang, Erin Dougherty, Gerardo Maupomé, Nannette Stamm, Brian Shue

**Affiliations:** 1 School of Public Health, San Diego State University, San Diego, California, United States of America; 2 Institute for Behavioral and Community Health, San Diego, California, United States of America; 3 Department of Psychology, San Diego State University, San Diego, California, United States of America; 4 San Diego State University/University of California San Diego Joint Doctoral Program in Clinical Psychology, San Diego, California, United States of America; 5 School of Medicine, University of California San Diego, La Jolla, California, United States of America; 6 School of Dentistry, University of California-San Francisco, San Francisco, California, United States of America; 7 Department of Bioengineering, School of Medicine, Stanford University, Stanford, California, United States of America; 8 School of Communication, San Diego State University, San Diego, California, United States of America; 9 University of New Mexico, Albuquerque, New Mexico, United States of America; 10 El Rio Health, Tuscon, Arizona, United States of America; 11 Fairbanks School of Public Health, Indiana University, Indianapolis, Indiana, United States of America; 12 Vista Community Clinic, Vista, California, United States of America; 13 Innercare, Brawley, California, United States of America; Hamadan University of Medical Sciences School of Dentistry, IRAN, ISLAMIC REPUBLIC OF

## Abstract

This paper describes the simultaneous co-development of Oral Health Behavior Social Support (OHBSS) scales in English and Spanish. OHBSS scales assess social support for toothbrushing, flossing, and dental care utilization, which are targets for interpersonal-level interventions to promote oral health among Hispanic/Latino adults. The focus was on Mexican-origin adults, who comprise the largest United States Hispanic/Latino subgroup and experience a high oral disease burden. All participants self-identified as Mexican-origin adults (ages 21–40 years old), living along the California-Arizona-Mexico border. Independent samples were recruited for each study partnering with Federally Qualified Health Centers. First, we conducted semi-structured interviews about social support for oral health behaviors in August to November 2018 (Study 1, N = 72). Interviews were audio recorded, transcribed (in original language, Spanish or English), and qualitative data were coded and analyzed in Dedoose following three topical codebooks; excerpts were used to co-create the large bilingual item data bank (OHBSSv1). The item bank was pre-tested via 39 cognitive interviews between December 2019 to March 2020, reviewed by an expert panel with several bilingual members, reduced to 107 Spanish/109 English items (OHBSSv2), then pilot tested in January to December 2021 (Study 2, N = 309). Pilot survey data were analyzed through Exploratory Factor Analysis and Horn’s parallel analysis, overall and by language, to examine response patterns and inform item selection (OHBSSv3). The scales queried social support for toothbrushing, flossing, and dental care utilization across 39 items from three sources (family, health providers, others/friends), plus up to nine optional dental care-related items (Study 3, conducted April 2022 to February 2023, N = 502). Confirmatory Factor Analysis (CFA) assessed model fit, overall and by language (multiple group CFA). Final OHBSS scales include 37 items, plus seven optional items. Acceptable model fit for three-factor structures for each oral health behavior was found, providing evidence of the scales’ construct validity. Cronbach’s alphas and McDonald’s omegas were tabulated; all were above 0.95, overall and by language, supporting scales’ internal consistency.

## Introduction

Disparities in oral diseases and access to care persist among racial/ethnic minority adults in the United States (US), particularly among individuals of lower socioeconomic status (SES), on Medicaid, and those living in rural geographic locations [1]. Hispanics/Latinos/as/x/e comprise the largest racial/ethnic group in the US [2]. They are of diverse heritage [3], with the largest subgroup of US Latinos being of Mexican-origin, either US-born or Mexico-born, with many living along the Mexico border region in California (CA) and Arizona (AZ) [4]. Mexican-origin adults are disproportionately disadvantaged with unmet dental needs, and Mexican immigrants had worse oral health status than other Latino heritage groups [5–7]. Mexican-American adults have the highest periodontal disease prevalence compared to non-Hispanic Whites and non-Hispanic Blacks [1,8]. Poor oral hygiene practices are a potentially modifiable behavioral risk factor for worse oral health.

Improving and maintaining daily oral hygiene for Mexican-origin adults can help reduce risk of oral diseases. Hygiene behavior frequency affects clinically assessed oral health outcomes; e.g., dental caries and periodontal disease [9–13]. The American Dental Association (ADA) recommends twice daily toothbrushing and once daily flossing, and annual dental visits [14,15]. Brushing alone does not clean interproximally (i.e., in between teeth) and many do not floss effectively ([9]). Longitudinal clinical trials showed good plaque control effectively ameliorates the rate of periodontal disease progression [16–18]. Good oral hygiene may effectively manage early periodontal disease [19]. Mexican-American adults may face barriers to proper adherence to ADA hygiene recommendations, such as knowing how to properly brush and floss [20]. Understanding how factors like social support could be targeted in future interventions to promote good oral hygiene are needed for Mexican-origin adults.

Dental visits provide opportunities for oral hygiene instruction and education about preventing oral diseases. However, US adult dental care utilization has been declining nationwide (41% to 37% in 2001–2010) [21]. Lack of insurance and high cost are frequently cited barriers to dental care among Hispanic immigrants [22]. There are challenging structural and financial barriers to accessing dental care, but some aspects of the complex health system could be navigated with support from others. There is strong evidence for family system [23], peer support [24] and community health worker interventions [24,25] to successfully promote behavior change, including among US Hispanics/Latinos [26]. Thus, sources and types of social support for oral health-promoting behaviors are important components for behavioral interventions. Yet, no domain-specific social support measure exists for oral health.

Social support refers to a social relationship with another individual from whom they might draw some support (such as a family member, friend, or health provider, for example). Social support has both structural (e.g., number of social ties with others, frequency of contacts) and functional components (satisfaction with emotional, informational, appraisal and instrumental support) [27,28], with the structural component as a necessary antecedent to social support. Perceived availability of structural and functional components is often measured [29–31], and important for health [31]. Greater perceived social support is positively associated with better mental and physical health outcomes [32–37]. There is evidence of health benefits from social support interventions, including those involving Mexican heritage populations in the US [38], however, we do not have a full understanding of mechanisms of action [39]. Social support has been more frequently studied as a psychosocial resource that people can draw on to help them cope with stressors or problem solve barriers [40,41]. Two dominant theories about how social relationships and social support influence health include: a) a direct model through physiological processes (like inflammation) and b) an indirect model affecting behaviors, which then influence the physiological process and health outcomes [41–43].

Research on social support in oral health is growing, though the existing body of research is still mostly descriptive, and conducted using varied brief general social support scales [44–49]. Dahlan and colleagues recently reviewed the relationship between social support and several oral health outcomes among immigrants, and found higher social support positively associated with dental visits and oral hygiene behaviors [44]. Existing validated general social support scales do exist [30,31], and most assess perceived functional support and emotional support. However, general scales may miss social support dimensions that are meaningful for particular diseases or behaviors [50]. For instance, in Sallis and colleagues’ diet and exercise social support measure, general social support measures were not associated with the domain-specific measure developed, and only the domain-specific social support measure was associated with the target heath behaviors [51].

Creating new Oral Health Behavior-specific Social Support (OHBSS) scales will enable more precise and accurate measurement for use in behavioral intervention research. The OHBSS scales were developed for Spanish- and English-speaking Mexican-origin adults, to assess their social support in the context of three oral health behaviors: toothbrushing, flossing, and dental care. For each behavior, we measure social support from three different sources: family, health providers, and others/friends. This paper details the development and refinement of a new tool to measure social support (the OHBSS scales) in English and Spanish. We present model fit statistics (overall and by language) and internal consistency coefficients to demonstrate the structural validity and reliability of the final scales.

## Materials and methods

### Dual-focus bilingual scale development approach and study design overview

This multi-phased, mixed methods sequential study [52] began with an exploratory qualitative study (Study 1) to co-create a large item bank for the scales in two languages (English and Spanish) simultaneously, employing a dual-focus approach informed by Erkut [53,54]. Study 2 employed qualitative cognitive interviews to pre-test items, then quantitatively analyzed the pilot survey draft scales, to attain a highly focused reduction in the size the scales. Study 3 employed quantitative methods to refine and test the structural validity and internal consistency of the final scales, and comprise the main final scale results [55,56]. In the present manuscript, we report results for the three studies.

**Fig 1:**
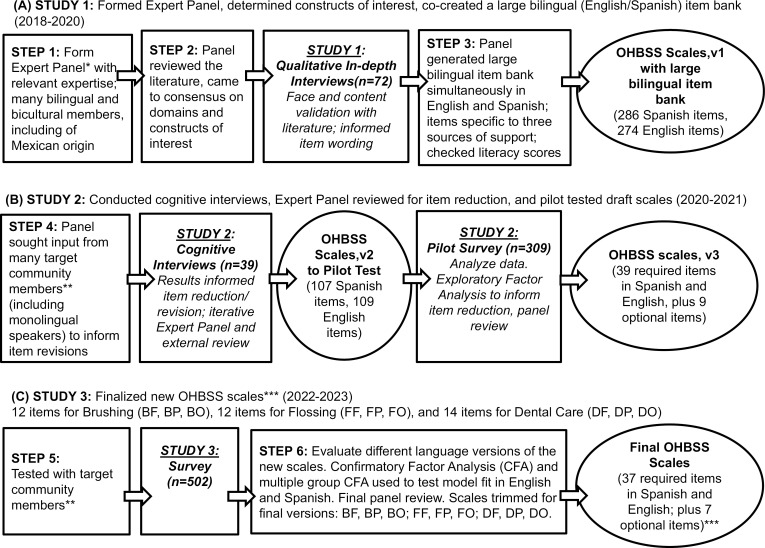
Dual-focus approach to developing bilingual (English/Spanish) Oral Health Behavior Social Support (OHBSS) scales. (A) Scale development steps to create v1 item bank and scale structure; (B) Steps to refine v2 items, reduce items for v3; (C) Validation phase to finalize new scales*Expertise: Knowledge of culture and language of target population, and relevant constructs of interest (social support, oral hygiene, dental care utilization). Types of social support: informational, instrumental, emotional, appraisal. Dual-focus approach informed by Erkut 2010, Erkut et al 1999.**Target community members:  Mexican-origin adults ages 21-40 years, living in urban or rural US southwestern counties near Mexico border. Sample characteristics monitored: Language preference (English/Spanish); sex (male/female); marital status (single/married)***Final OHBSS scales: Social support for Brushing (B), Flossing (F) and Dental Care (D) were assessed from each source of support: Family (F), Health Providers (P) and Others/Friends (O). Plus 7 optional Dental Care support items: translation, transportation, payment, and worries.

We first composed a multi-disciplinary study team of investigators with appropriate expertise and background in oral health, social support, biostatistics, translation, and psychology (specifically in scale development and validation). Some investigators and many study staff were bilingual in English/Spanish, and most of the staff were of Mexican origin. All investigators and several study staff, along with staff from the partner Federally Qualified Health Centers (FQHCs) and community, were part of the expert panel. The panel was knowledgeable about the priority populations, and many were fluent in and had an in-depth understanding of Mexican Spanish. We planned and conducted primary data collection through three studies to develop and refine the OHBSS scales (see Fig 1), outlining the dual focus approach to developing the bilingual OHBSS scales.

### Setting

Our community-engaged study was conducted in partnership with three FQHCs along the California (CA) and Arizona (AZ) border with Mexico (MX). Two large migrant FQHCs in CA, Vista Community Clinic (VCC) and Innercare (formerly *Clínicas de Salud del Pueblo, Inc., or CSDSP,* during Studies 1 and 2), were subcontracted partners involved in all phases of study planning and implementation. The largest FQHC in AZ, El Rio Health, partnered on Study 3.

### Priority population

We focused on Mexican-origin men and women, ages 21–40 years. We intentionally focused on the largest racial/ethnic group in the US, Hispanics/Latinos, and further selected to focus on adults of Mexican-origin background (the largest Hispanic/Latino heritage group). Clinical reasons supported our narrowed focus on Mexican-origin adults; they experience a high burden of disease, and poorer oral health than other Hispanic/Latino subgroups and non-Hispanic Whites and Blacks [1,6,8]. Periodontal disease prevalence trends among US adults over age 30 show the increase with age is largely driven by those in the moderate periodontal disease category; those in the mild or severe categories appeared stable over age 40 [57]. The target age range was selected for developmental reasons. “Late adolescence” (ages 18–20) is marked by significant physical, social, emotional growth and transitions in home, school, and work [58]. Age 21–40 was selected as it captured all “young adulthood.” This age range also encompasses a prime window of opportunity for behavioral intervention and preventive efforts that could significantly affect oral health [59]. All data collection activities involved both US- and MX-born young adults living near the southwestern US-Mexico border.

#### Eligibility criteria.

For all three studies, participants were eligible if they: 1) self-identified as Mexican-origin (defined broadly, included Mexican, Mexican-American, Chicano/a, and other identities), 2) were between 21–40 years old (at eligibility screening), 3) able to speak, read, and provide written informed consent in English or Spanish, and 4) resided in designated areas. Geographic inclusion criteria were expanded with each subsequent study to simplify screening and recruitment, more effectively reach new potential participants and enable enrolling independent samples to meet increasing sample size goals for each study. For Study 1, participants had to reside in northern San Diego County, CA, within specified clinic service areas near VCC’s largest clinic in Vista, or in Imperial County, CA, within Innercare’s clinic service areas. For Study 2, participants could reside anywhere in San Diego or Imperial counties. For Study 3, participants could reside anywhere in San Diego, Riverside, Orange, or Imperial Counties in CA (two CA-MX border counties, plus two counties where VCC and Innercare operated clinic sites); or Pima, Cochise, Santa Cruz, or Yuma Counties in AZ (all four AZ-MX border counties). Recruitment and data collection for Studies 2 and 3 occurred during the COVID-19 pandemic; it was simpler to define and screen residential inclusion eligibility criterion by county.

Exclusion criteria for all studies included: being edentulous or having a health condition requiring pre-medication before dental exams or having a mental or physical impairment and thus unable to provide informed consent. For women, being pregnant was a temporary exclusion criterion, due to pregnancy-related hormone changes that affect women’s oral health [60].

#### Study design balance characteristics.

For all three studies, each data collection effort recruited independent parallel samples of participants. Given our bilingual scale development goal, we closely monitored and strived to manage enrollment to achieve optimally balanced study samples, especially by language. The goal was for each sample to comprise about 50% Spanish-speakers (top priority), and ideally also 50% male (for sex-stratification analyses), and 50% married/partnered (to account for potential differences in available social support); also, this approach optimized statistical power to examine differences based on such characteristics. Thus, it was possible for an eligible and interested potential participant to not be enrolled, if a particular subgroup had already reached the target goal. In these cases, potential participants were waitlisted and contacted for a later study in this effort to achieve ideal cross-classified balanced samples by language, sex, and marital status in all phases.

#### Recruitment.

For all three studies, we employed convenience and snowball sampling to reach potential participants. We trained clinic partners’ community health workers (CHWs) to share study information and recruit potential participants. Being registered patients with our FQHC partners was not an eligibility criterion. However, to support recruitment efforts, VCC and Innercare sent text messages and/or called patients appearing to meet inclusion criteria to invite them to contact the study staff to enroll. Clinic CHWs recruited widely, and distributed paper flyers at various events (e.g., food distributions, back to school events, health fairs, dad’s club meetings) and shared electronic versions of flyers and short recruitment videos on their social media platforms (primarily Facebook and Instagram). In rural Imperial County, CA, outreach and recruitment efforts relied more on newspaper and radio ads, public service announcements, phone calls, and door-to-door and group presentations. Participants were also asked to share study information by word-of-mouth and recruit other potentially eligible friends or family members.

Study staff independently recruited, hosted booths at community and cultural events, gave presentations, and shared flyers with other community organization leaders. Leaders’ organizations included county oral health coalitions and health departments, hospital/health systems, language and trade schools for adults, grade schools and after-school programs to reach parents, cultural events (e.g., Día de Los Muertos and tamale festivals), and other settings (e.g., YMCA, laundromats, Mexican restaurants and grocery stores, libraries). For most in-person recruitment events, dental kits or single flossers with study contact information were distributed.

#### Ethical considerations.

The San Diego State University (SDSU) Institutional Review Board (IRB) reviewed and approved each of the three studies (Study 1: HS-2017-0351; Study 2: HS-2019-0117; Study 3: HS-2021-0201). Written informed consent was obtained in English or Spanish before data collection for each study. A copy of the grant application and scope of work for the University of California San Francisco (UCSF) subcontract was also submitted to the UCSF IRB, which reviewed UCSF co-investigators’ roles, and approved their roles on the study annually between 2018–2021, then deemed that annual UCSF IRB renewal was no longer required after 2021 (IRB#18-24606). All the clinics relied on the SDSU IRB. All participants received a copy of the fully executed document, which included a certificate of confidentiality. Paper consent forms were used for Study 1, while consent signatures were obtained electronically in Studies 2 and 3. Only trained study staff consented and enrolled participants. Study 3 procedures were also reviewed and approved by the National Institute of Dental and Craniofacial Research (NIDCR) and a NIDCR-appointed Clinical Study Oversight Committee (CSOC). A NIDCR-appointed external monitoring contractor reviewed all informed consent forms, regulatory documents, and data from a subset of participants.

## Data collection and analysis procedures

### Study 1: Qualitative interviews

Investigators conducted a social support and oral health literature review to inform writing a semi-structured interview guide in English to be administered with the target population of Mexican-origin young adults. The literature review was conducted by systematically searching in the following databases: PubMed, CINAHL, Web of Science, and PsycInfo. Relevant references cited in the studies were also reviewed. A recent existing review of social support measures by Lopez and Cooper [30] and a social support theory and measurement text by Lakey and Cohen [29] were also closely reviewed at this phase. The investigators determined that the interviews should solicit information about oral hygiene (all methods of cleaning teeth, mouth, and gums) and all experiences related to seeking all types of dental care (preventive, restorative, and emergency services). The interview guide was translated into Spanish independently by three professional translators, each with appropriate experience and understanding of Mexican Spanish and who were briefed on the study topic and purpose. The three translations were independently reviewed, compared, discussed with the translators, and adjudicated over multiple iterations to ensure conceptual and linguistic equivalence in meaning and tone. The guide was then finalized by one experienced bilingual/bicultural co-investigator, following evidence-based methods to maximizing equivalency across scales [61]. This step was critical, as these translations affected how the primary constructs of interest for the scale were phrased in the interview guides (see S1 Table).

Semi-structured interviews and interviewer-administered brief demographic and health behavior surveys were conducted with 72 participants in August-November 2018 by trained interviewers. Interviews lasted about 1.5 hours, were audio-recorded, and conducted in-person in research or clinic offices, or a private community space (e.g., library room). Participants received $25 cash and a dental kit. Audio recordings of the interviews were transmitted to one of three professional translators/transcribers via secure file transfer protocol (ftp) for transcription. Interviews were transcribed in their original language (English or Spanish). Two of the three transcribers were familiar with the project and involved in the interview guide translation process. Interviewers reviewed their transcripts for accuracy, clarified any inaudible sections (referring to audio files if necessary), de-identified text, then added fieldnotes and other administrative information to the final, clean transcript file.

#### Study 1 Data Analysis.

Qualitative coding and analysis was facilitated by Dedoose (Version 8.1.8), a secure cloud-based program which allows for layered co-coding (i.e., the coding of two concepts applied to one excerpt) [62]. Coding was guided by three detailed codebooks that captured all domains of interest and were designed to be overlapping for cross-coding analysis (see Table 1 for codebook overview, and full codebooks in S2 Table): 1) oral health behaviors; 2) sources of support; and 3) types of social support (pre-defined codes from the social support literature). Emergent codes were developed for the first two codebooks after reading a subset of interview data. The codebooks and all codes applied were in English. Six bilingual coders completed about fourteen hours of trainings in qualitative methods and interviewing [63], coding in Dedoose, and study-specific coding procedures. Coders were assigned codebooks, and coded the same passages in order to become certified to code independently, after reaching at least 80% agreement across code applications. All coders used a tracking sheet to raise questions about code applications. These were reviewed at least weekly by the project manager (PM) and principal investigator (PI), then discussed at coder meetings, and the code definitions and examples in the codebooks were clarified as needed to ensure all coders coded consistently. All coding and quality checks were done on a rolling basis until completed in June 2019; over 20,000 excerpts were coded. Each coder completed a second review to double check all their final code applications. Then one independent trained coder performed quality checks and reviewed all code applications on all transcripts. There were very few instances of any missing codes or disagreements about codes applied at this final checking stage. When there was a missing or disputed code, that excerpt was reviewed by the PM, discussed with the PI, and the final decision made was documented. It was determined that no additional or new codes were needed, and saturation had been reached.

**Table 1:** Overview of qualitative codebooks, by topic.

Codebook Title (number of codes)	Description of codes
1) Codebook 1: Oral Health Behaviors (17 codes)	Participants’ past and present oral hygiene behaviors, and utilization and access to different types of dental care services (e.g., preventive, restorative, emergency), including changes over time, barriers, motivations, and occasional oral health practices.
2) Codebook 2: Sources of Social Support (33 codes)	Participants’ interactions with people and organizations as well as the context (e.g., time period like “childhood”) and modes of the interactions (including different types of communication channels).
3) Codebook 3: Types of Social Support (36 codes)	Positive and negative influential factors of engaging or not engaging in oral health behaviors, and multiple types of social support (instrumental, informational, emotional, appraisal).

Dedoose is a mixed methods program and allows for co-coding of an excerpt with multiple concepts. Three general codes were also applied to all sections to capture valence (positive/facilitator or negative/barrier) and illustrative quotes. Study design characteristics were included in Dedoose as “descriptors” (labels, not codes) to enable sorting and comparison by language, sex, and marital status of participant, as well as study site. Full codebooks are available in S2 Table.

**Table 2:** OHBSSv1 scale instructions, definitions, format, structure and example in English and Spanish.

**Survey: Dental Health Support** Please rate each statement 3 times using an “X” to mark your answer, think about where you receive dental treatment/care (U.S. and/or Mexico). 1) First, think about help or support you get from your FAMILY/ For the first column mark how much support you receive from your FAMILY 2) Then think about help or support you get from HEALTH PROVIDERS/ For the second column mark how much support you receive from your HEALTH PROVIDERS 3) Then think about any OTHER PEOPLE/ For the third column mark how much support you receive from any OTHER PEOPLE The response options indicate the frequency of the help or support you receive from the three groups mentioned above, where “1” is the lowest frequency and “5” is the highest frequency. There is a “N/A” option if the question does not apply for a specific or any group. Most importantly, there are no right or wrong answers, and your name will not be included in this survey. Please look at the example below:																																				
For each statement, how much of this type of support do you get from family, healthcare providers and other people?	**FAMILY** (includes immediate and extended family members you may or may not live with)													**HEALTH PROVIDERS** (includes medical providers, dentists and dental specialists)												**OTHER PEOPLE** (includes anyone that is not family or a health provider)										
	**Never** ^1^		**Rarely** ^2^		**Sometimes** ^3^			**Often** ^**4**^	**Always** ^**5**^			**N/A** ^**6**^		**Never** ^1^		**Rarely** ^2^	**Sometimes** ^3^			**Often** ^**4**^		**Always** ^**5**^		**N/A** ^**6**^		**Never** ^1^		**Rarely** ^2^	**Sometimes** ^3^			**Often** ^**4**^		**Always** ^**5**^	**N/A** ^**6**^	
*EXAMPLE:* They make sure I get my flu shot.								X								X										X										
**Encuesta: Apoyo con la Salud Dental** Por favor marque cada pregunta 3 veces usando una “X” para indicar su respuesta, piense sobre donde recibe su tratamiento/cuidado dental (E.U.A y/o México) 1) Primero, piense sobre la ayuda o apoyo que recibe de su FAMLIA/ Para la primera columna marque cuanto apoyo recibe de su FAMILIA 2) Después, piense sobre la aynda o apoyo que recibe de sus PROVEEDORES DE SALUD/ Para la segunda columna marque cuanto apoyo recibe de sus PROVEEDORES DE SALUD 3)Luego, piense sobre cualquier OTRA GENTE/ Para la tercera columna marque cuanto apoyo recibe de cualquier OTRA GENTE Las opciones indican con qué frecuencia recibe ayuda o apoyo de los tres grupos mencionadoes anteriormente, donde el “1” es la frecuencia más baja y el “5” es la frecuencia más alta. Si la pregunta no es applicable para un grupo específico o ninguno de los grupos, marque la opción de “N/A.” Sobre todo, no hay respuestas correctas o incorrectas y sun ombre no se incluirá en esta encuesta. Por favor mire el ejemplo a continuación:																																				
Para cada frase,¿cuánto apoyo recibe de su familia, proveedores de salud y otras personas?		**FAMILIA** (incluya miembros de familia con los que pueda o no vivir)											**PROVEEDORES DE SALUD** (incluya proveedores médicos, dentistas y especialistas dentales)												**OTHER PEOPLE** (incluya cualquier persona que no sea familiar o un proveedor de salud)											
		**Nunca** ^**1**^		**Raramente** ^**2**^		**A Veces** ^**3**^	**A Menudo**_**4**_			**Siempre**_**5**_	**N/A**_**6**_		**Nunca** ^**1**^		**Raramente** ^**2**^			**A Veces** ^**3**^	**A Menudo**_**4**_		**Siempre**_**5**_		**N/A**_**6**_		**Nunca** ^**1**^		**Raramente** ^**2**^			**A Veces** ^**3**^	**A Menudo**_**4**_		**Siempre**_**5**_			**N/A**_**6**_
*EJEMPLO: Se aseguran de que reciba mi inyecci*ó*n para la gripe*.							X								X										X											

Code occurrences in text excerpts were tabulated and summarized in reports generated by Dedoose as part of an initial content analysis to understand the qualitative data excerpts and begin to look for meaningful themes and patterns. Next, co-code occurrence reports were generated in Dedoose to examine excerpts with multiple layered codes of interest applied. These reports illustrated layered inter-relationships of interest, showing narratives about different types of social support (Codebook 3) provided by different sources (Codebook 2) for each target oral health behavior (Codebook 1). These co-code reports aided our thematic analysis. We sorted excerpts by Codebook, to identify potential patterns across the layered co-coded excerpt for each behavior. A subgroup of the expert panel (PI, PM, and co-investigators with qualitative expertise) conducted thematic analyses, and led discussions with the full expert panel to guide decisions about the new scale structure and content.

The investigators were interested in all themes, including less frequent themes, to understand the full range of types and sources of social support for the target oral health behaviors. Themes were summarized and informed discussions and decisions about the scale’s intended content and purpose. Critical guiding themes included the time point (current versus past) and people; the panel decided to focus the analysis and scale creation on current patterns of social support provided from people, as opposed to support from other sources. Among people, we separated them into groups of family members, health professionals, and others. For oral hygiene, the panel decided to focus on brushing and flossing behaviors only. We inquired about rinsing and other teeth/mouth cleaning behaviors, but given the lack of ADA guidelines about rinsing, these were not retained as priority target behaviors to include in the new scale. There were three themes identified related to dental utilization: access to care, routine dental care, and major dental treatment. The co-coded reports were sorted by these themes and text excerpts from each group of reports were analyzed and used to construct potential items for the item bank.

#### Scale structure.

The initial structure and response options for the OHBSS scales are presented in Table 2. This structure came directly from the qualitative interviews, because participants talked about different people being sources of support; thus, it became clear that we needed to ask about sources of support for each oral health behavior separately. Instructions and definitions of target behaviors were written by the investigators, in English and Spanish. Participants are asked to rate each social support item three times, once for each source of support (family, health providers, others/friends). A frequency response scale asked how often support was received from each source (1 = Never to 5 = Always, or 6 = not applicable). A non-oral health example was provided to show that each item would need to be rated three separate times.

#### Item generation.

An expert panel comprised of all nine investigators, two bilingual study staff, and two bilingual clinic partner staff (thirteen total) met repeatedly in 2019 to generate items. English and Spanish items were generated simultaneously by the panel to create the new scales’ item bank, using interview quotes/excerpts as the source material for item wording. Items were originally drafted in either English or Spanish, then cross-language versions were immediately considered, rather than creating a scale in one language and then translating to the other upon completion. During the panel’s bi-directional item generation group process, items were also checked for equivalence in both languages. The panel purposefully included repetitive items with alternate wording options. The panel reviewed items to ensure wording for each was clear, concise, and did not have a double negative and was not double-barreled. Per the dual-focus approach, panel meetings were conducted bilingually; additionally, the panel held three monolingual Spanish-speaking sessions with clinic CHWs who preferred and/or only spoke Spanish to review the Spanish item bank.

### Study 2: Cognitive interviews, expert panel review, and pilot testing

Cognitive interviews are a useful step in developing new scales [64,65], and typically a minimum of ten interviews, or 30 interviews conducted iteratively, are recommended to identify problematic items [66,67]. During the cognitive interview, trained bilingual staff asked a series of probes to prompt respondents to “think aloud” and state in their own words what question items were asking, aiming to have them verbalize their thought process in why they selected a particular response option, and explain what the response meant. This process checked for understanding of each item as intended, assessed acceptability and face validity with the target population, and informed item reduction and revisions. Interviewers documented feedback about problematic items (specifically, needing to repeat a question or possible response options, or when terms were not understood or needed alternate explanation). Overall, we captured diverse opinions in each language (English/Spanish), from both sexes (male/female), and in different sites (urban/rural) through 39 interviews and covered the entire OHBSSv1 item bank. Participants received $20. Interviews were conducted in-person from December 2019 to March 2020; after the COVID-19 pandemic started, interviews shifted to virtual webconferencing platforms.

The expert panel expanded beyond the core group of thirteen at this stage, to include more input from bilingual study staff, clinic staff, and community members in the review of all cognitive interview feedback to inform item reduction and refinement. The panel referred to a list of criteria for evaluating items and documented reasons for the decision to drop or retain items. Literacy scores were considered in the decisions for item retention. Flesch-Kincaid [68] scores were tabulated for English items, and comparable Fernandez-Huerta [69] scores were tabulated for Spanish items to assess approximate reading ease/grade level of each item. Additionally, the panel balanced comprehensive construct coverage (all four types of social support assessed for each of the three oral health behavioral targets in both languages), with efficiency (reducing the number of domains to most essential, and combining similar domains where possible), considerations for application (ensuring that domains are measured that will be needed for future use), and respondent burden (the scale must be user-friendly and not too burdensome in terms of difficulty or time to complete). The panel also ensured that parallel items in the brushing and flossing sections were retained. The refined item bank had three sections, one for each behavior of interest (one section about brushing, one section about flossing, and one section about dental care utilization with different subsections). The OHBSSv2 item bank for pilot testing (not shown) included 107 items in Spanish and 109 items in English (including alternate English wording for two related hygiene items).

A final round of four virtual cognitive interviews (two in English, two in Spanish) was conducted to time administration of the OHBSSv2 scale, assess respondent fatigue and burden, and provide a final check before launching Study 2.

#### Pilot testing.

The OHBSSv2 scales had 107 Spanish and 109 English items. Due to the COVID-19 pandemic, data collection was carried out online and the OHBSSv2 survey was programmed into REDCap Academic [70,71]. Pilot survey data were collected January-December 2021. Participants received a $30 Amazon gift card for completing the one-time, one-hour survey (n = 340). The minimum sample size determination for this phase was guided by results of a measure development review indicating subject-to-item ratios ≥ 2 [72].

#### Study 2 data analysis.

Pilot survey data were cleaned and analyzed for panel review and item reduction. Frequency distributions and patterns of responses were explored. The panel dropped participants who did not complete at least 50% of the OHBSS scale items with valid answers; 31 of the 340 replied majority “not applicable” responses and were dropped. However, prior to finalizing the analytic sample, patterns of not applicable responses were reviewed more closely. For example, several reported not flossing and did not answer or selected not applicable for the flossing section but answered the rest. To maximize available data, participants were retained for analyses if they completed selected sections of the OHBSS scales (i.e., participants who answered brushing but not flossing items were retained for the brushing scale analyses). There were 279 participants who answered all OHBSS sections; 298 completed Brushing, 302 completed Flossing, and 287 completed Dental Care sections.

We used a series of exploratory factor analysis (EFA) and maximum likelihood extraction methods with orthogonal varimax rotation to identify scale factors [73,74] and item factor structure. Items with factor loadings ≥ 0.4 were retained for review by the expert panel [73,75–77]. Orthogonal rotation is a recommended approach that facilitates results interpretation [78,79]. Analyses were run separately for each behavior (brushing, flossing, and dental utilization), overall and by language, with alternate sets of items to ascertain if factors loaded more clearly with certain items dropped. Many sets of iterative EFA results (not shown) were reviewed closely by the panel in tandem with item descriptive frequencies, especially for similar items with alternate wording, to identify the best items to keep. From the distributions of descriptive frequencies, it was clear that social support varied by source of support group (family, health providers, others/friends). EFAs were run for each behavior and source of support group combination. The panel examined the English- and Spanish-language EFA results to check for similar patterns in factor loadings to see if items loaded in the same way in both language groups. The panel also considered which items were essential for the new scales in terms of importance for capturing changes in social support in a future intervention and were most relevant for the target behaviors, as well as if the set of items covered all desired types of social support.

We also used Horn’s parallel analysis [80] to confirm the estimated factor structure, overall, and by language. Horn’s parallel analysis results also guided decisions about factor retention by comparing observed eigenvalues to those from a computer-generated set of random variables with the same number of scale items and subjects [77,81–83]. We retained eigenvalues that exceeded the 95^th^ percentile in the parallel analysis [82,83]. Analyses were conducted in SAS (v9.4, SAS Institute, Cary, NC).

#### Scale refinement and item reduction.

The expert panel met regularly from December 2021 to March 2022 to review results from the iterative EFAs and Horn’s parallel analyses, identify factor structure, and make decisions to further refine the scale and reduce items to create the OHBSSv3. Items were dropped if the item content was redundant, and/or the item had problematic or less preferred phrasing, and/or did not load well (≥0.4). The panel also considered the importance of an item for use in a future behavioral intervention (e.g., will it capture potential changes in social support for oral health behaviors) in deciding to retain or drop items. Overall scale length, respondent burden, and literacy levels of items were considered.

After scale refinement and item reduction, cognitive interviews with the shortened OHBSSv3 scales were administered and timed with four participants from the community (two in English, two in Spanish) as a final check for clarity before launching the Study 3 survey.

### Study 3: Finalizing scales

#### Data collection.

The online Study 3 survey with the 39 required plus nine optional items in the OHBSSv3 scales was conducted between April 2022 and February 2023, following the same recruitment and online survey administration procedures as Study 2. Participants received a $30 Amazon gift card after completing the survey (N = 540). In this phase, we again referred to our prior sample size determination criteria [72], but increased it significantly higher than our Study 2 goals to ensure a much larger final analytic sample size for scale validation purposes.

#### Study 3 data analysis.

Using SPSS (v29, IBM Corp., Armonk, NY), descriptive statistics were calculated for the OHBSSv3 scales. Descriptive statistics included means, standard deviations, modes, medians, skewness, and kurtosis, or frequencies and percentages, as appropriate. Data were tabulated to examine the distribution of item responses and extent and mechanisms of missingness. Of 540 participants, 502 provided complete responses to the OHBSSv3 scales. Two participants had a few missing OHBSSv3 scale responses and regression imputation was used to maximize available data for analysis.

Using MPLUS version 8, a series of confirmatory factor analysis (CFAs) were conducted to evaluate the structural validity of the nine OHBSSv3 scales (required items only) by behavior-source of social support group combination, overall, and by language group (n = 199 Spanish-speakers, n = 303 English-speakers). While there is no consensus on CFA sample size recommendations, CFAs can adequately be undertaken with a minimum sample of 200 [84]. The first CFA examined the fit of a three-factor structure that represented toothbrushing social support, with the subdomains of social support from family (BF), health providers (BP), and others/friends (BO). The second CFA examined the fit of a three-factor structure that represented flossing social support, with the subdomains of social support from family (FF), health providers (FP), and others/friends (FO). The third CFA examined the fit of a three-factor factor structure that represented dental care social support, with the subdomains of social support from family (DF), health providers (DP), and others/friends (DO).

The maximum likelihood robust estimator was used as the CFA estimation method. Several recommended goodness-of-fit indicators were applied to assess model fit [85]. Statistical model fit was evaluated through the Satorra-Bentler $\chi^{2}$ (S-B $\chi^{2}$) for multivariate non-normal data [86]. S-B $\chi^{2}$ indicates adequate statistical fit if the estimate was non-significant at alpha 0.05. Descriptive fit was also evaluated, considering that S-B $\chi^{2}$ is sensitive to sample size and can falsely reject an adequate model. The comparative fit index (CFI) [87], the root mean square error of approximation (RMSEA) [88]. and the standardized root mean residual (SRMR) [89] were also employed to evaluate descriptive fit. CFI  ≥  0.90 indicates acceptable model fit and CFI  ≥  0.95 indicates good model fit. RMSEA and SRMR values  ≤  0.08 indicate acceptable model fit, with  ≤  0.05 indicating good model fit. Acceptable model fit was indicated if at least two of the three descriptive fit estimates (CFI, RMSEA, SRMR) indicated adequate descriptive fit [89]. Acceptable statistical and/or descriptive fit supported adequate structural validity for the corresponding set of OHBSS scales.

An exploratory phase was planned if a CFA resulted in unacceptable model fit for any of the OHBSS scales. The first step was to inspect the CFA modification fit indices, which suggest changes to the model that could substantially improve model fit. The suggested changes would be applied only if they were theoretically reasonable, practical, and that could help achieve acceptable model fit. However, if modification indices suggested complex, unexpected changes, the planned follow-up step was to conduct EFA to examine the content of suggested factors and loading of items. It was expected that EFA findings would result in changes (e.g., trimming of items) to the model that would improve CFA model fit. The goal was to present final sets of OHBSS scales with adequate structural validity as measured by adequate statistical and/or descriptive model fit.

Using R Studio (v4.0.3 (2020-10-10), Boston, MA), Cronbach’s alphas and McDonald’s omegas were calculated to examine the internal consistency of the OHBSS scales. Bootstrapped 95% confidence intervals (CIs) were also calculated for each estimate of internal consistency. Cronbach’s alpha is the dominant measure of internal consistency, but this estimate has assumptions that are often unlikely to be met [90]. The estimate can also be inflated due to a large sample size and large number of items. McDonald’s omegas were calculated because they are more robust to deviation from assumptions, and can provide a more accurate estimate of internal consistency.

## Results

Participant demographics for each study’s independent sample are in Table 3, and summarize distribution across age groups (21–30, 31–40), self-identified biological sex (female, male), marital status (married, single), language preference (Spanish, English), and site (CA or AZ county). Desired balance across the three key design characteristics (language, sex, marital status) was achieved in Studies 1 and 2. Study 3 was not as evenly balanced by sex, and skewed to female. Language stratification was prioritized over the other two study design characteristics (sex, marital status) in Study 3 to meet sample size goals on the study timeline. While there were more English-speakers than Spanish-speakers in Study 3, each language group was large enough to support stratified analyses by language, a critically important feature for scale development and validation in both languages.

**Table 3:** Participant characteristics.

	Study 1 N = 72	Study 2 N = 309 *	Study 3 N = 502
	N (%)	N (%)	N (%)
Age			
21–30 years old	32 (44)	182 (59)	267 (53)
31–40 years old	40 (56)	127 (41)	235 (47)
Sex			
Female	39 (54)	178 (58)	397 (79)
Male	33 (46)	131 (42)	105 (21)
Marital Status			
Married	35 (49)	143 (46)	188 (63)
Single	37 (51)	166 (54)	314 (37)
Language preference			
Spanish	39 (54)	167 (54)	199 (40)
English	33 (46)	142 (46)	303 (60)
Site			
San Diego County, CA	32 (44)	187 (61)	262 (52)
Imperial County, CA	40 (56)	122 (39)	96 (19)
Riverside County, CA	N/A	N/A	38 (8)
Orange County, CA	N/A	N/A	18 (3)
AZ counties	N/A	N/A	88 (18)

* Does not include the 39 cognitive interview participants, or the 8 participants who completed the timed surveys before widely launching OHBSSv2 and OHBSSv3.

AZ counties included: Pima, Cochise, Santa Cruz, and Yuma.

### Study 1 results

The qualitative phase yielded the working first draft item bank for the scales (OHBSSv1), with a total of 274 English items and 286 Spanish items (see Table 4), to be pre-tested for refinement and item reduction with input from community members prior to broader pilot testing (full item bank not shown). Items were generated in five categories: dental care access, routine dental care (including preventive care and simple procedures), major dental treatment, brushing, and flossing.

**Table 4:** Summary of initial bilingual item bank for OHBSSv1, by theme.

Language	A) Dental Care Access items	B) Routine Dental Care items	C) Major Dental Treatment items	D) Brushing items	E) Flossing items	Total items
English	66	62	20	69	57	274
Spanish	72	68	21	68	57	286

Alternately worded versions of items were drafted, so there are not necessarily equivalent numbers of items in each section.

OHBSSv1 appeared to have face and content validity. The OHBSSv1 scale structure and content was similar to other validated general social support scales found in the literature and used in health research. The initial OHBSSv1 queried social support from three different potential sources of social support (family, health providers, and others/friends). The OHBSSv1 item bank also included items that appeared to assess the intended social support dimensions of interest (i.e., informational, instrumental, emotional, and appraisal types of social support). Table 5 summarizes the OHBSSv1 item bank content by type of social support.

**Table 5:** Summary of initial bilingual item bank for OHBSSv1, by type of social support.

	A) Dental Care Access items	B) Routine Dental Care items	C) Major Dental Treatment items	D) Brushing items	E) Flossing items	Total items
English						
Instrumental	29	32	15	19	16	111
Informational	24	23	1	29	18	95
Emotional	11	7	4	13	14	49
Appraisal	2	0	0	8	9	19
Total English items	66	62	20	69	57	274
Spanish						
Instrumental	32	36	16	16	14	114
Informational	26	25	1	31	20	103
Emotional	12	7	4	14	15	52
Appraisal	2	0	0	7	8	17
Total Spanish items	72	68	21	68	57	286

Alternately worded versions of items were drafted, so there are not necessarily equivalent numbers of items in each section.

### Study 2 results

Feedback from the cognitive interviews assisted in identifying the best items to retain. The final round of cognitive interviews prior to Study 2 pilot testing suggested face and content validity of the OHBSSv2 scales. The OHBSSv2 scales in the pilot test survey included 107 Spanish items and 109 English items, and had three sections to cover the three target behaviors (Table 6) and four types of social support (Table 7).

**Table 6:** OHBSSv2 scale item bank overview, by behavior.

Behavior	English * items	Spanish items
Brushing	28*	27
Flossing	18*	17
Dental Care	63	63
TOTAL	109*	107

* There were 109 items total in English, including alternately worded versions for “nag me” in the oral hygiene brushing and flossing sections. This is the unique item count.

Note participants rate each item three times, once for each source of support: 1) Family, 2) Health Providers, 3) Others/Friends.

**Table 7:** OHBSSv2 scale item bank, by type of social support.

Social Support domain	Brushing items	Flossing items	Dental Care items	TOTAL items
Instrumental	4	3	22	29
Informational	14	8	24	46
Emotional	4	4	13	23
Appraisal	5	2	4	11
TOTAL*	27*	17*	63*	107*

* This is the unique item count, and does not include the 2 alternately worded instrumental hygiene items in English.

Note participants rate each item three times, once for each source of support: 1) Family, 2) Health Providers, 3) Others/Friends.

#### Factor structure identification.

Patterns of EFA results were highly suggestive that brushing, flossing, and dental care social support each had a three-factor structure. Initial EFA results ranged from three to seven factors in the Full Sample, though all items primarily loaded on the first three factors, following a clear pattern for groups by three sources of support for family, health providers and others/friends (see S3 Table: Study 2 EFA item loadings). Brushing and dental care behavior results yielded more than three factors. When additional factors were identified, they included very few items that had cross-loaded, but with lower loadings on the fourth-seventh factors. Patterns suggested a three-factor structure, and generally persisted in each language group as well. Generally, consistent patterns emerged across the three behaviors, with three factors for each of the different source of support groups. There did not appear to be separate factors to delineate different types of social support. The panel interpreted these patterns to be supportive of a three-factor structure. Parallel analysis results also showed that items loaded together into a three-factor structure for brushing, flossing and dental care social support, with a factor for each of the three source of support groups. Additional EFAs were run for each behavior with restrictions to three factors, and all items loaded cleanly and grouped as expected across the three source of support groups. The ranges of item loadings for each behavior, overall and by language, are summarized in Table 8. The panel interpreted the three-component results as supportive of brushing, flossing, and dental care social support each having a three-factor structure.

**Table 8:** EFA item loading ranges, overall and by language.

FAMILY (F)	FULL SAMPLE	ENGLISH	SPANISH
Brushing (B)	0.64–0.82	0.59–0.82	0.60–0.85
Flossing (F)	0.66–0.87	0.60–0.86	0.70–0.87
Dental Care (D)	0.62–0.77	0.59–0.79	0.62–0.78
**HEALTH PROVIDERS (P)**			
Brushing (B)	0.63–0.84	0.58–0.83	0.66–0.84
Flossing (F)	0.60–0.87	0.57–0.85	0.59–0.90
Dental Care (D)	0.56–0.79	0.51–0.81	0.53–0.81
**OTHERS/FRIENDS (O)**			
Brushing (B)	0.72–0.85	0.66–0.84	0.73–0.85
Flossing (F)	0.69–0.88	0.71–0.88	0.66–0.90
Dental Care (D)	0.61–0.82	0.61–0.83	0.54–0.86

From the EFA and Horn’s parallel analysis results, there was support for distinct factors for each source of support group, leading to structuring separate OHBSS scales separately, for a total of nine scales for each behavior-source of support combination.

#### Scale refinement.

The panel made one substantial change to the scales’ structure after reviewing the frequency distribution of item responses. There were certain types of barriers related to accessing dental care services that were common for a large group of respondents, but not applicable to everyone. The panel conducted additional sensitivity analyses (not shown) to understand the differences between “never” and “not applicable” responses. Patterns of responses were similar, and the panel decided to collapse the two response categories. The panel recognized “never” is different than “not applicable,” but agreed to drop the “not applicable” option, and updated the instructions to include “not applicable” with the “never” category. Thus, the response options for the OHBSSv3 scales were: 0 = Never (or “not applicable”), 1 = Rarely, 2 = Sometimes, 3 = Often, 4 = Always.

Eleven parallel brushing and flossing items were retained. One general hygiene item was kept, “They remind me to get more dental supplies (for example, toothbrush, toothpaste, floss, etc.)”/ “Me recuerdan de conseguir más productos dentales (por ejemplo, cepillo, pasta, hilo dental, etc.)”. This general dental supply item is only asked once, but is scored with both toothbrushing and flossing, thus, there are 12 items scored for toothbrushing and 12 items scored for flossing. This general hygiene item is only counted once in the item count.

Scales for each behavior are denoted as follows: Brushing (B; 12 unique items), Flossing (F; 11 unique items, plus one general dental supplies item gets scored with both brushing and flossing) and Dental care (D; 16 unique items). There are a total of 39 required items to compute scale scores for each behavior. From among the 39 items, one item assessed support for dental hygiene supplies and was included in the subscale score for both Brushing and Flossing. Each behavioral scale can assess social support from three different sources of support: Family (F), Health Providers (P), and Others/Friends (O). Thus, there are nine OHBSSv3 scales, named with the combination of the oral health behavior and source of support (e.g., “Brushing Social Support from Family,” abbreviated “BF”). All the scales included items for informational, instrumental, emotional and appraisal types of social support, although overall, they skewed informational.

When the panel reviewed the dental care items most frequently rated as “not applicable,” they decided to move nine items into a supplemental, optional set of questions. These items queried relevant topics related to dental care and captured aspects of social support that the panel wanted to assess, but these did not fit into the required scales as constructed. These nine dental care items were deemed important, but not generally applicable to everyone. Short screening questions were added by the panel to identify if the individual needed help for one of four optional topics: 1) language interpretation services (one item), 2) transportation (one item), 3) payment (three items), or 4) relieving dental fears/worries (four items). If applicable, respondents can complete up to nine additional items related to seeking dental care. See S4 Table for a copy of the OHBSSv3 scales, with 39 required items plus up to nine optional items, in English and Spanish. Table 9 summarizes the number of items in the OHBSSv3 scales by behavior, and Table 10 displays the breakdown of the number of items in the OHBSSv3 scales by type of social support.

**Table 10:** OHBSSv3 scale items, by type of social support.

Social Support domain	Brushing (B) items	Flossing (F) items	Dental Care (D) items	Main Scales TOTAL	Trans-late* item	Trans-port* item	Pay* items	Worry* items	MAX TOTAL (+Optional) items
Instrumental	3	3	6	12	1	1	1	0	15
Informational	7	6	7	20	0	0	2	0	22
Emotional	1	1	1	3	0	0	0	4	7
Appraisal	1	1	2	4	0	0	0	0	4
TOTAL**	12	11	16	39	1	1	3	4	48

* Optional item(s), if applicable.

** Note participants rate each item three times, once for each source of support: 1) Family (F), 2) Health Providers (P), 3) Others/Friends (O).

**Table 9:** OHBSSv3 scale items, by behavior.

Behavior	Items
Brushing (B)	12
Flossing (F)	11*
Dental Care (D)	16
MAIN SCALES TOTAL	39**
OPTIONAL DENTAL CARE ITEMS	
Translation	1
Transportation	1
Payment	3
Worries	4
OPTIONAL ITEMS TOTAL	9
MAXIMUM POSSIBLE TOTAL	48**

* 1 brushing item that asked about social support for providing all dental supplies is asked once, but is used to compute the Brushing social support scale score and is also scored with the Flossing social support scale.

** Participants rate each item three times, once for each source of support: 1) Family (F), 2) Health Providers (P), 3) Others/Friends (O).

The OHBSSv3 scales took 17-22 minutes to complete, and all participants indicated the instructions, questions and response options were clear. Feedback from the cognitive interviews suggested the OHBSSv3 scales were clear and understandable, and had acceptable face and content validity.

### Study 3 results

#### Structural validity

##### Overall sample.

**Fig 2:**
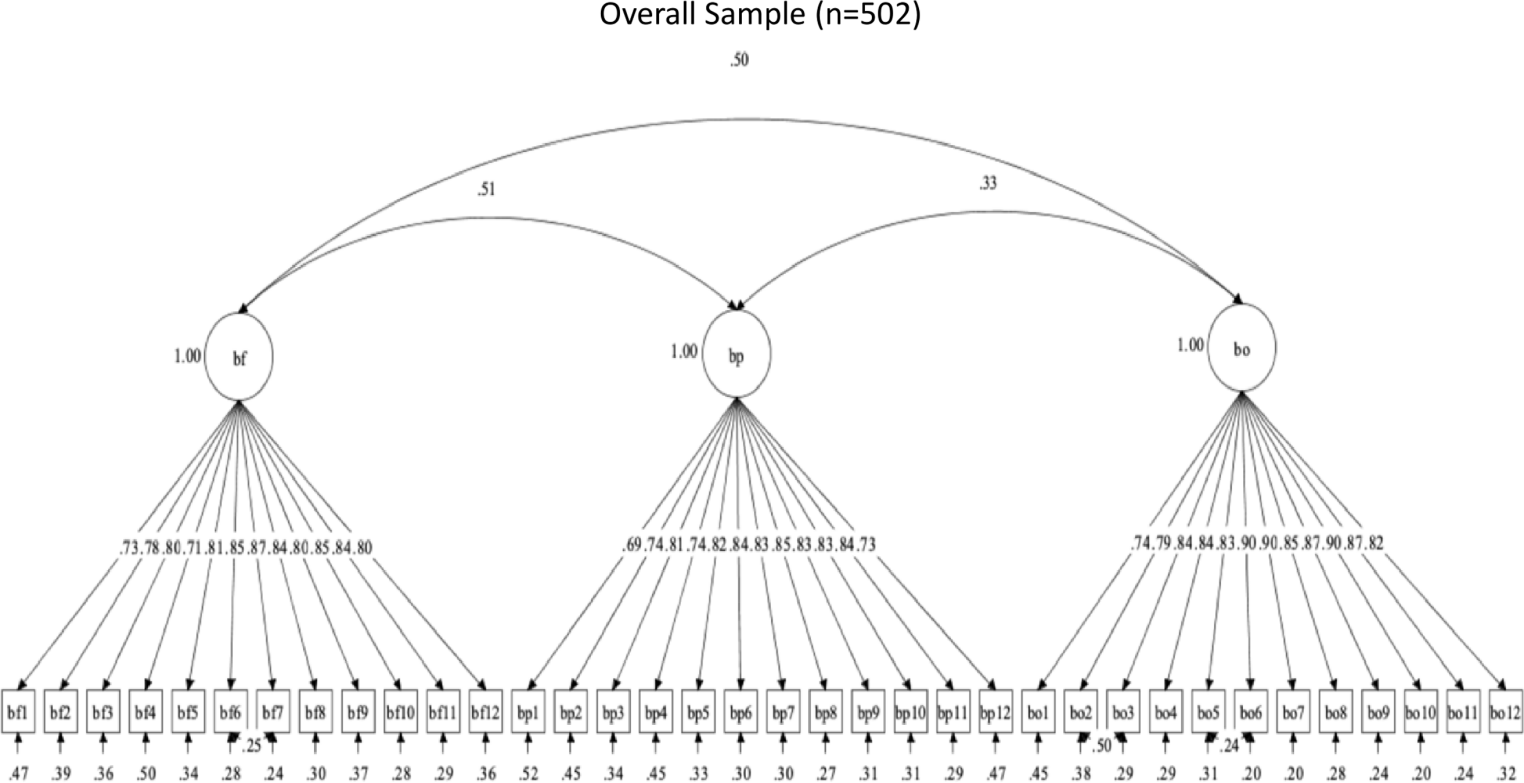
Factor structure for Brushing social support scales.

**Fig 3:**
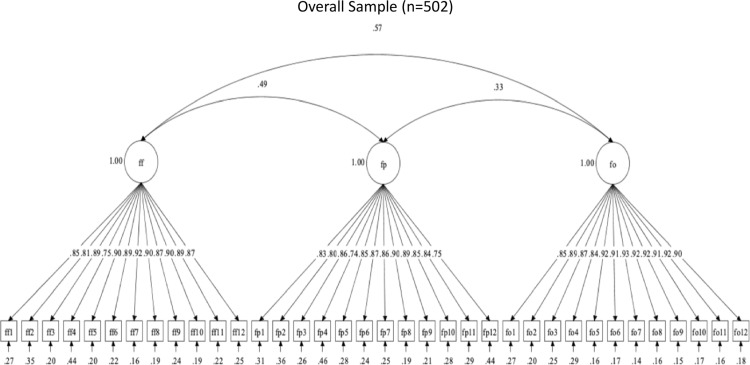
Factor structure for Flossing social support scales.

**Fig 4:**
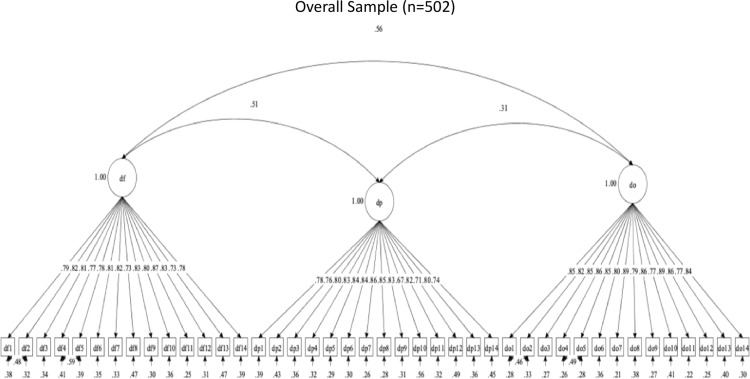
Factor structure for Dental Care social support scales.

The OHBSSv3 scales had 39 required items. Three CFAs evaluated the structural validity of the OHBSSv3 scales in the total sample for Study 3. For toothbrushing, flossing, and dental care social support, descriptive fit supported a three-factor structure (see Table 10). Toothbrushing social support had the subdomains of social support from family (BF), health providers (BP), and others/friends (BO) (Fig 2). Flossing social support had the subdomains of social support from family (FF), health providers (FP), and others/friends (FO) (Fig 3). Dental care social support had the subdomains of social support from family (DF), health providers (DP), and others/friends (DO) (Fig 4). Statistical fit was poor in all CFAs. These findings support adequate structural validity for each set of three scales among Mexican-origin adults, even without adequate statistical fit.

##### By language group.

**Fig 5:**
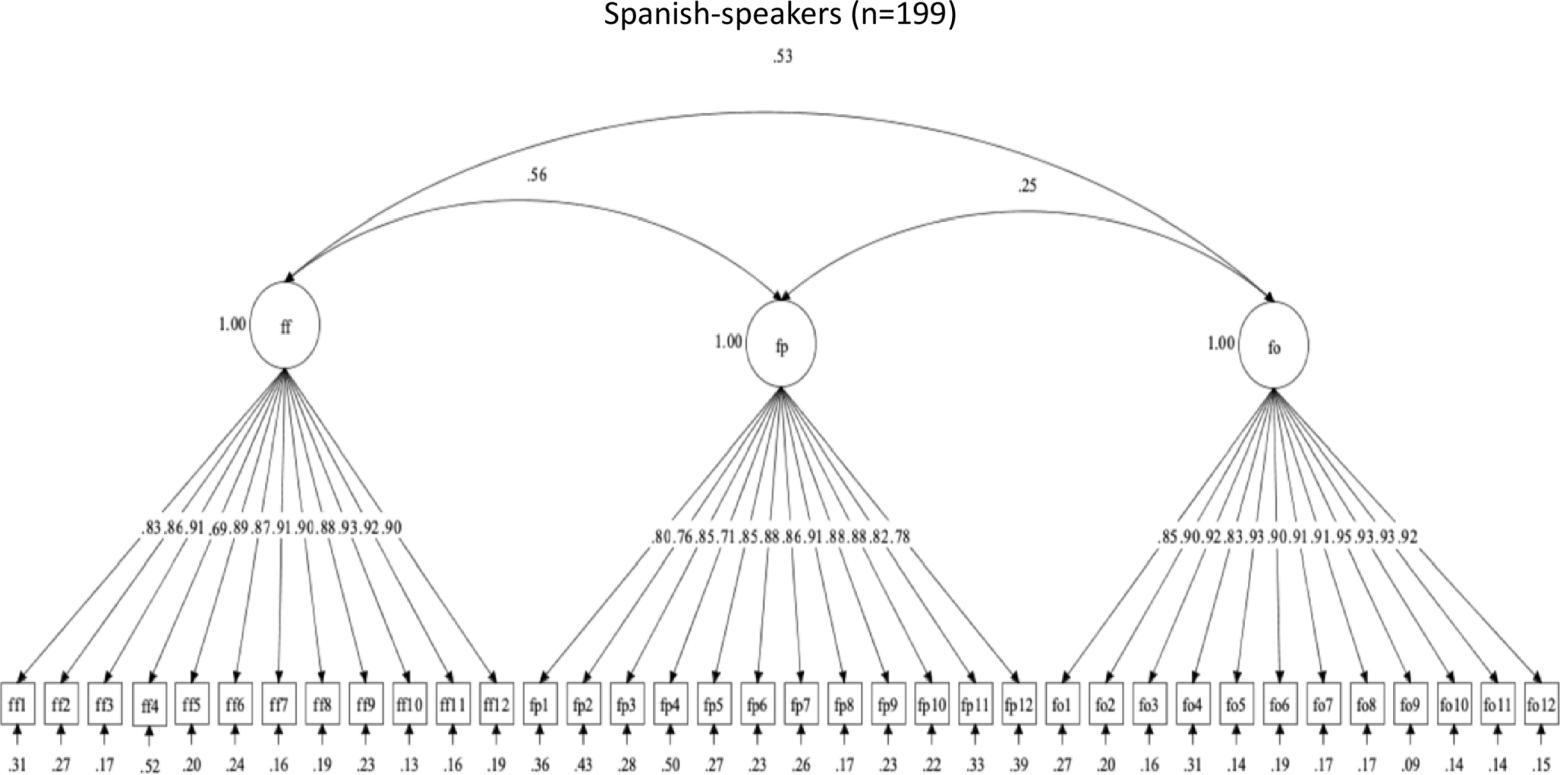
Factor structure for Flossing social support scales in Spanish.

**Fig 6:**
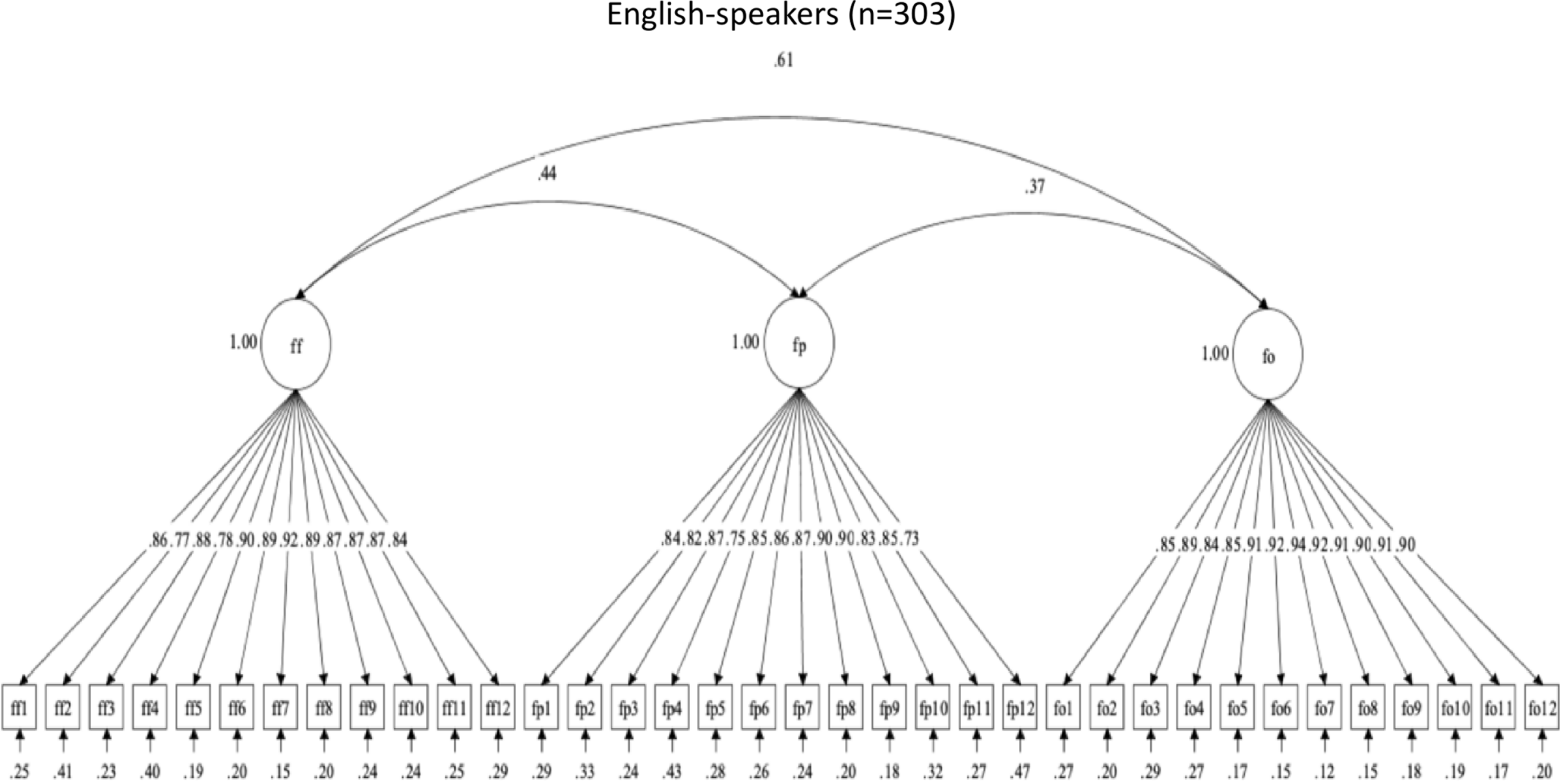
Factor structure for Flossing social support scales in English.

Follow-up CFAs evaluated the structural validity of the OHBSSv3 scales by Spanish-speaking and English-speaking Mexican-origin adults. Model fit indices are summarized in Table 11. Only the set of flossing social support scales (FF, FP, FO) had adequate structural validity in both language groups. The three-factor structure for flossing social support had adequate descriptive fit but poor statistical fit in both language groups. The flossing social support scales were not revised. Figs 5 and 6 show the flossing social support scales’ factor structures and item correlations, by language.

**Table 11:** Confirmatory factor analysis model fit indices.

	Chi-Square Test	CFI	RMSEA	SRMR	Minimal revisions to achieve acceptable model fit.^3^
Full sample (N = 502)					
**Three-factor Brushing**	**2246.156**	**0.876**	0.075 *	0.049 *	
**Three-factor Brushing (minor revisions)**	**2114.547**	**0.886**	0.072 *	0.049 *	**See changes below**
**Three-factor Flossing** ^1^	**1821.453**	0.916 *	0.064 *	0.046 *	
**Three-factor** **Dental Care** ^2^	**4004.364**	**0.845**	0.074 *	0.062 *	
**Three-factor** **Dental Care update**^1^ **(minor revisions)**	**2789.533**	**0.875**	0.070 *	0.061 *	**See changes below**
Spanish (n = 199)					
Three-factor Brushing	1439.831	0.843	0.085	0.062 *	
**Three-factor Brushing (minor revisions)**	**1310.223**	**0.867**	0.079 *	0.061 *	BO2 WITH BO3; BF6 WITH BF7; BO5 WITH BO6.
**Three-factor Flossing** ^1^	**1207.347**	0.909 *	0.072 *	0.051 *	
Three-factor Dental Care	2470.526	0.843	0.081	0.070 *	Suggested Follow-up EFA
Three-factor Dental Care update	1914.486	0.853	0.082	0.069 *	Dropped ^2^ problematic items
**Three-factor** **Dental Care update**^2^ **(minor revisions)**	**1737.809**	**0.876**	0.076 *	0.069 *	DF4 WITH DF5; DF1 WITH DF2; DO1 WITH DO2; DO4 WITH DO5
English (n = 303)					
**Three-factor Brushing**	**1661.316**	**0.876**	0.077 *	0.052 *	
**Three-factor Brushing (minor revisions)**	**1616.562**	**0.881**	0.076 *	0.051 *	BO2 WITH BO3; BF6 WITH BF7; BO5 WITH BO6.
**Three-factor Flossing** ^1^	**1576.419**	**0.897**	0.074 *	0.050 *	
Three-factor Dental Care	3201.518	0.821	0.081	0.066 *	Suggested Follow-up EFA
**Three-factor** **Dental Care update**	2456.842	0.832	0.081	0.065 *	Dropped ^2^ problematic items
**Three-factor** **Dental Care update**^1^ **(minor revisions)**	**2230.571**	**0.855**	0.076 *	0.064 *	DF4 WITH DF5; DF1 WITH DF2; DO1 WITH DO2; DO4 WITH DO5

^1^Flossing social support scale has 12 items; this includes the one general hygiene item that is also in the Brushing social support scale.

^2^Dental Care social support scale is for the final 14 item version.

^3^Item abbreviations: B = Brushing, D = Dental Care; F = Family, O = Others/Friends.

* indicates fit indices that show at least acceptable model fit.

CFI =  Comparative fit index;

RMSEA =  Root Mean Square Error of Approximation;

SRMR =  Standardized Root Mean Square Residual.

CFI values of at least 0.90 indicate acceptable model fit and values >  0.95 indicate good model fit.

SRMR and RMSEA values less or equal to 0.08 indicate acceptable model fit and values lower or equal to 0.05 indicate good model fit. Two out of the three descriptive fit indices need to show at least acceptable model fit to declare that a model has adequate descriptive model fit.

**Bolded** indicates acceptable model fit.

**Fig 7:**
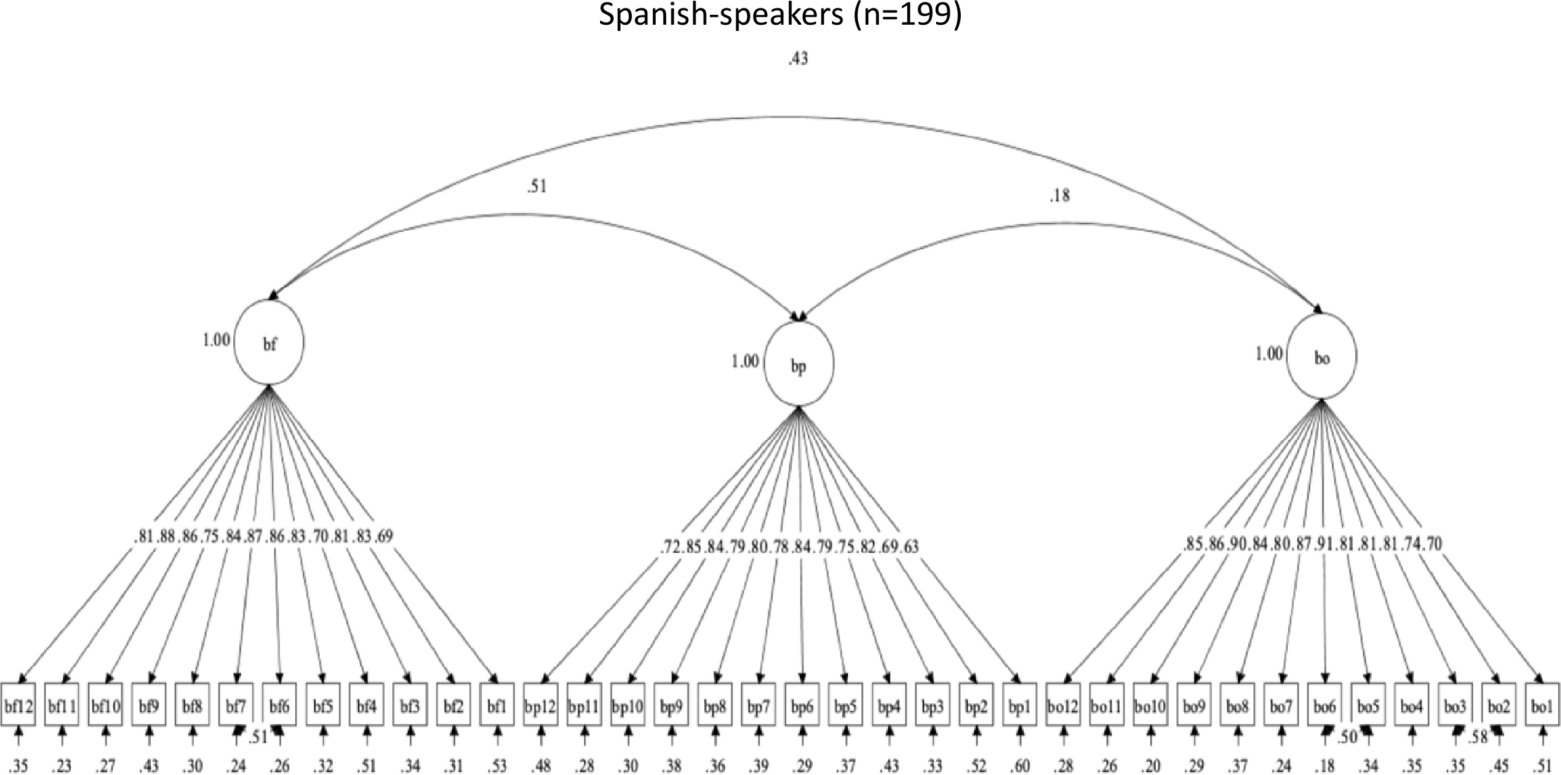
Factor structure for Brushing social support scales in Spanish.

**Fig 8:**
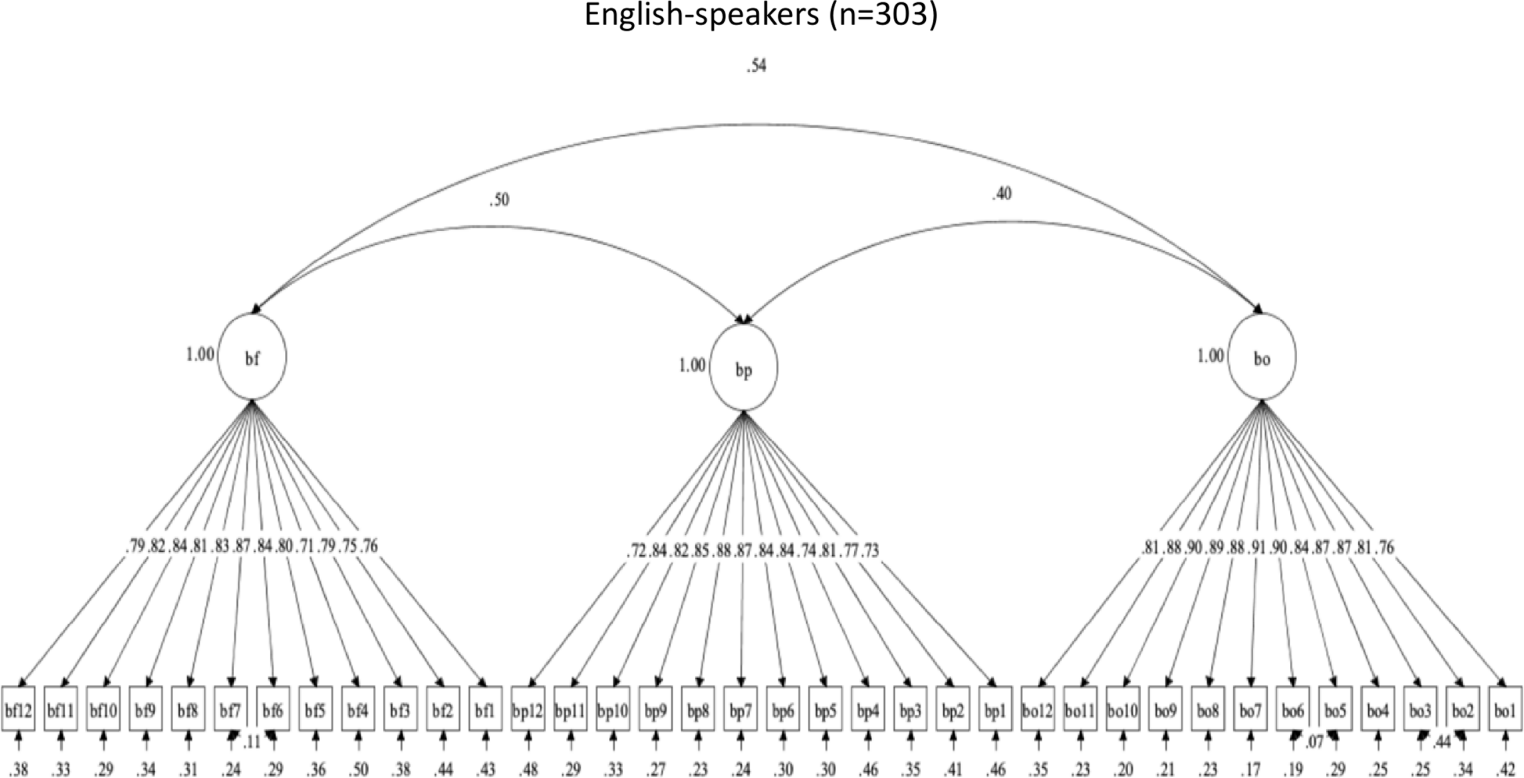
Factor structure for Brushing social support scales in English.

Revisions were made to the set of brushing scales because of inadequate structural validity across language groups. The three-factor structure for brushing social support initially had acceptable descriptive fit among English-speakers, but poor descriptive fit among Spanish-speakers. The modification indices suggested that the following items should be allowed to correlate to achieve acceptable descriptive fit in both language groups: Brush-Family (BF) items 6 with 7 in the BF scale; and Brush-Others (BO) items 2 with 3, and BO5 with BO6 in the BO scale. These were considered practical and substantive changes to the model; however, these changes did not result in changes to the item structure of the scales, and items from the same subscales should correlate. After these changes were made to the theoretical model, the BF, BP, and BO brushing social support scales had adequate descriptive fit in both language groups. Figs 7 and 8 show the brushing scales’ factor structures and item correlations, by language. The new findings support the structural validity of this set of brushing scales but with a slight change in the latent structure.

Revisions were made to the set of dental care scales because of inadequate structural validity across language groups. The three-factor structure for dental care social support had poor statistical and descriptive fit in both Spanish- and English-speaking Mexican-origin adults. The modification indices suggested complex, unexpected changes to the model to improve model fit (items should be predicted by a different factor or items from different scales should be allowed to correlate). Thus, a follow-up EFA was conducted to examine the content of suggested factors and loading of items.

Findings still supported the three-factor structure of dental care social support, with the subdomains of social support from family, health providers, and others/friends (DF, DP, DO, respectively). However, after closer inspection of factor loadings and item content, two items may have been problematic in the models. First, one item (“They helped me get care”/ “Me ayudan a conseguir cuidado dental”) was similar to and simpler than another item (“They helped me manage challenges or obstacles to getting dental care”/ “Me ayudan a solucionar problemas para poder tener cuidado dental”). The latter item was dropped. The dropped item also had a poor literacy score and was more difficult to understand. Another dental care item (“They talk me through my dental treatment options” / “Me hablan de las opciones que tengo para mi tratamiento dental”) was reviewed and deemed to have idiomatic phrasing in English; it also assumed a need for treatment with different potential options to be discussed that may not be applicable to everyone. This item was dropped. Model fit was improved after these two problematic items were dropped.

**Fig 9:**
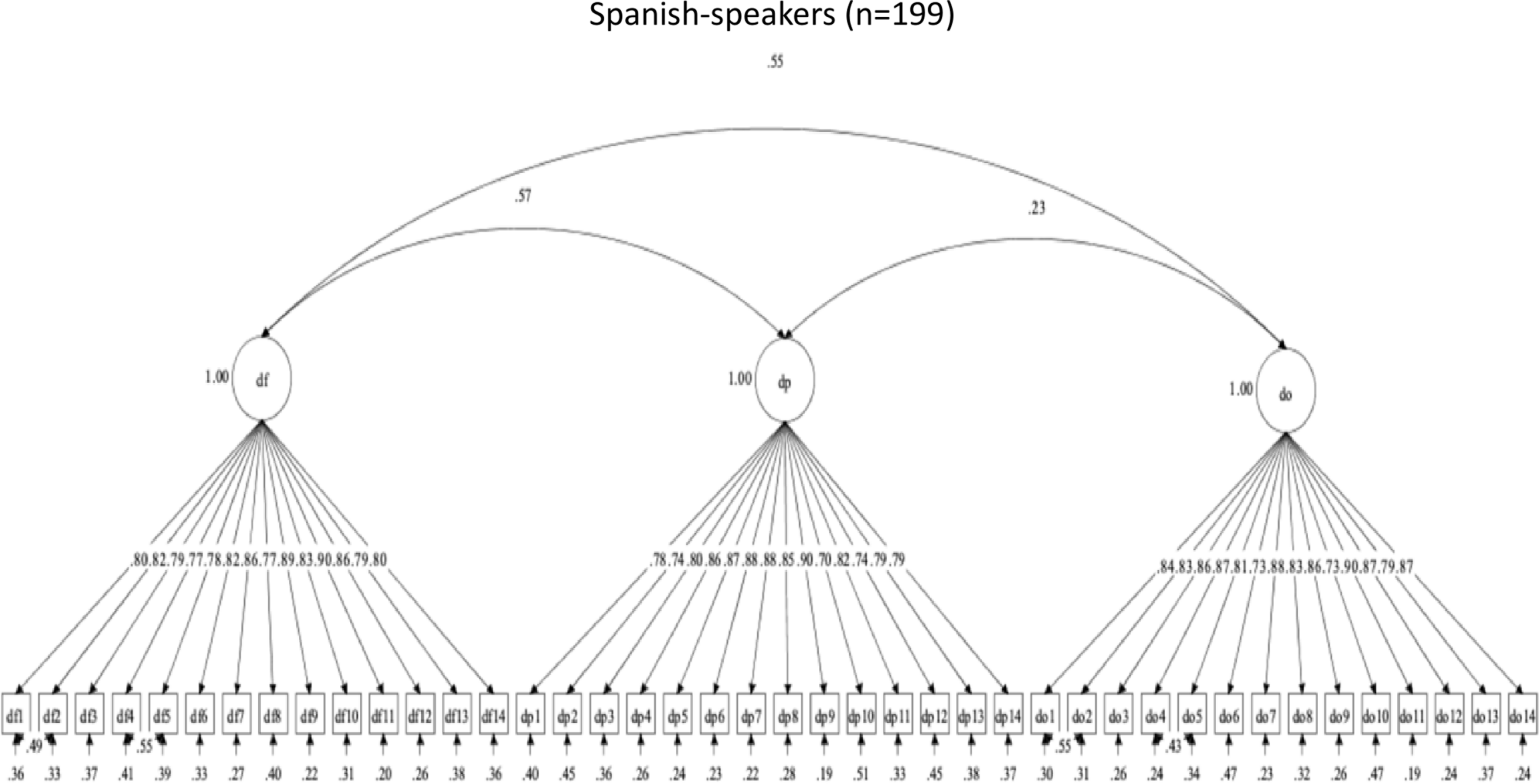
Factor structure for Dental Care social support scales in Spanish.

**Fig 10:**
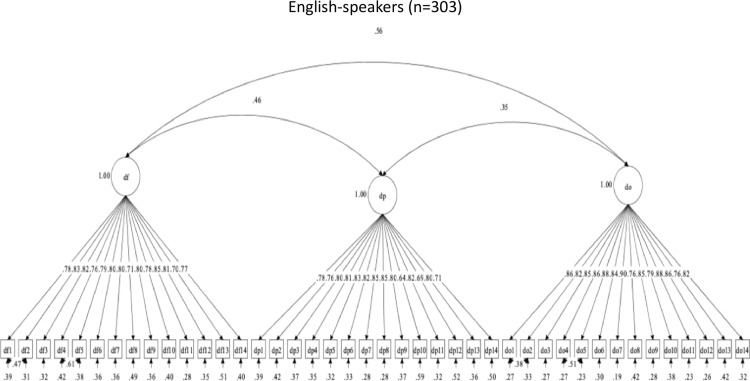
Factor structure for Dental Care social support scales in English.

A new CFA was conducted to examine the fit of a three-factor structure for dental care for social support without the two problematic items. This model still had poor statistical and descriptive fit in both language groups. However, the modification indices suggested that the following items should be allowed to correlate in the model to achieve acceptable descriptive fit in both language groups: Dental-Family items 1 with 2, and DF4 with DF5 in the DF scale; Dental-Others items 1 with 2, and DO4 with DO5 in the DO scale. These were considered practical and substantive changes to the model. They would not result in new changes to the structure of the scales, and items from the same subscale should correlate. After these changes were made to the theoretical model, the updated model had acceptable descriptive fit in both language groups for the Dental Care social support scales. Figs 9 and 10 show the dental care scales’ factor structures and item correlations, by language. The findings support the structural validity of the revised set of dental care scales.

These changes resulted in trimming two required dental care items from the final OHBSS scales. The updated models had adequate fit. The results suggested that these final versions of the OHBSS scales for brushing social support, flossing social support, and dental care social support have adequate structural validity, in the full sample, and by language.

### Final expert panel review and item reductions

The expert panel conducted a final close review of the items and the response distributions for the 37 required and nine optional items, following scale development best practices [55]. The panel noticed an inconsistency with items in the optional section, and decided to drop two of the four items in the optional dental worries section. For one item, the English and Spanish item wording did not exactly match, and it had a poor literacy score, so this item was dropped: “They listen to my fears about going to the dentist./ Escuchan mis preocupaciones sobre ir al dentista.” The panel missed this wording inconsistency before testing OHBSSv3. The expert panel decided to also drop the other optional item with the “dental fear” phrasing (“They ease my fears about dental treatments./ Calman mis miedos sobre los tratamientos dentales.”) and only retain the two items with the “dental worries” phrasing. The distribution of responses was similar across the four dental worries items, so the panel felt dropping these two optional items was worthwhile to create a more parsimonious set of final scales.

#### Final OHBSS scales.

The final OHBSS scales include 37 required items and up to seven optional items, with each unique item asked three times, once for each of three different sources of social support. The scales collectively cover three behaviors, three sources of social support, in two languages. See Tables in S5 for the final OHBSS scales.

Tables in S6 lists all required and optional items in the final OHBSS scales in English and Spanish side-by-side, along with information about type of social support and literacy level (Flesch-Kincaid scores, Fernandez-Huerta scores, and reading ease grade level ratings), and variable names. Our goal was to keep the final scales below 9^th^ grade reading levels, and ideally lower, closer to 5^th^ or 6^th^ grade levels (which approximates completing elementary school). Flesch-Kincaid scores for the English brushing and flossing scale items ranged from grade 0 (pre-Kindergarten) to 7.5, with most items written at or below a 5^th^ grade level. Fernandez-Huerta scores for the Spanish brushing and flossing scale items ranged from 0 to 6.0, with one item scoring at a more difficult level. Flesch-Kincaid scores for the English dental care items ranged from 0.5 to 7.3 among required items and from 4.1 to 9.6 among optional items. Fernandez-Huerta scores for most Spanish dental care items ranged from 4.0- to 8.0 among required and optional items, with one required item and three optional items scoring at a more difficult level. Scale instructions were at a 7^th^ grade level in both languages.

See Table 12 for a summary of the number of items in the final OHBSS scales by behavior, and Table 13 for the final OHBSS scales by types of social support.

**Table 12:** Final OHBSS scales, by behavior.

Behavior	Items
Brushing	12 *
Flossing	11 *
Dental Care	14
OHBSS SCALES TOTAL	37**
OPTIONAL DENTAL CARE ITEMS (include only if applicable)	
Translation	1
Transportation	1
Payment	3
Worries	2
MAXIMUM ADDITIONAL OPTIONAL SCALE ITEMS	7
MAXIMUM POSSIBLE TOTAL	44**

* 1 item that asked about social support for dental supplies is asked once, but is scored with both the Brushing and Flossing social support scales.

** Participants rate each item three times, once for each source of support: 1) Family, 2) Health Providers, 3) Others/Friends.

**Table 13:** Final OHBSS scale, by type of social support.

Social Support domain	Brushing items	Flossing items	Dental Care items	Main Scale TOTAL	Trans-late * item	Trans-port * item	Pay * items	Worry * items	MAX TOTAL (+Optional) items
Instrumental	3	2	6	11	1	1	1	0	14
Informational	7	7	7	20	0	0	2	0	22
Emotional	1	1	1	3	0	0	0	2	5
Appraisal	1	1	0	2	0	0	0	0	4
TOTAL**	12	11	14	37	1	1	3	2	44

* Optional item(s), if applicable.

** Participants rate each item three times, once for each source of support group: 1) Family, 2) Health Providers, 3) Others/Friends.

The response distributions of each required and optional item in the final OHBSS scales are presented in S7 Tables, summarizing the descriptive mean, standard deviation, median, mode, skewness and kurtosis. The optional items applied to some Study 3 participants as follows: Help was needed for translation services when seeking dental care by 112 of the 502 participants (22%); transportation help was needed by 69 of the 502 participants (14%); almost half (242 of the 502 participants) identified needing help paying for dental care; and dental worries were reported by 172 of the 502 participants (34%).

The final OHBSS Brushing, Flossing, and Dental Care social support scale scores by source of support are summarized in Table 14. For each behavior-source of support combination, the scale is scored by averaging the required items in that section. Behavioral social support scale scores varied by source of support, with health providers having the highest scores on average, reflecting that they provided more perceived social support for all behaviors. On average, family social support scores were slightly lower than the health provider group for all behaviors. Others/friends had very low average scores, indicating this group rarely provided social support for any oral health behaviors. Social support scores were similar for brushing and dental care, and usually slightly higher than for flossing. Overall, Spanish-speakers had slightly higher average OHBSS scores than English speakers. Significant differences by language were found for the BF, FF, DF and BP and DP social support scales.

**Table 14:** Final OHBSS scale scores by source of social support, overall and by language.

	Full sample (N = 502)			English sample (N = 303)			Spanish sample (N = 199)		
	Mean (SD)	Median (Mode)	Skewness (Kurtosis)	Mean (SD)	Median (Mode)	Skewness (Kurtosis)	Mean (SD)	Median (Mode)	Skewness (Kurtosis)
**Family (F)**									
Brushing * (BF)	2.2 (1.3)	2.3 (4)	−0.2 (−1.2)	2.1 (1.3)	2.1 (4)	>−0.1 (−1.3)	2.5 (1.2)	2.7 (4)	−0.6 (−0.8)
Flossing * (FF)	1.8 (1.3)	1.8 (4)	0.2 (−1.3)	1.7 (1.3)	1.5 (0)	0.3 (−1.3)	2.0 (1.3)	2.1 (4)	−0.04 (−1.4)
Dental Care * (DF)	2.1 (1.2)	2.2 (4)	−0.1 (−1.2)	2.0 (1.2)	2.1 (4)	<0.1 (−1.2)	2.2 (1.2)	2.4 (4)	−0.3 (−1.0)
**Health Providers (P)**									
Brushing * (BP)	2.8 (1.1)	3.1 (4)	−1.0 (−0.1)	2.7 (1.2)	3.0 (4)	−0.8 (−0.4)	2.9 (1.0)	3.2 (4)	−1.2 (0.8)
Flossing (FP)	2.7 (1.2)	3.1 (4)	−0.8 (−.5)	2.7 (1.2)	3.0 (4)	−0.8 (−0.6)	2.8 (1.2)	3.2 (4)	−0.9 (−0.4)
Dental Care * (DP)	2.8 (1.1)	3.1 (4)	−1.0 (.2)	2.7 (1.1)	2.9 (4)	−0.8 (−0.1)	3.0 (1.06)	3.4 (4)	−1.3 (1.1)
**Others/Friends (O)**									
Brushing (BO)	0.9 (1.1)	0.4 (0)	1.2 (0.3)	0.9 (1.2)	0.3 (0)	1.3 (0.4)	1.0 (1.1)	0.6 (0)	1.0 (0.1)
Flossing (FO)	0.7 (1.1)	0.2 (0)	1.5 (1.2)	0.8 (1.1)	0.2 (0)	1.6 (1.3)	0.7 (1.0)	0.1 (0)	1.4 (0.9)
Dental Care (DO)	1.0 (1.1)	0.6 (0)	1.2 (0.4)	0.9 (1.2)	0.4 (0)	1.2 (0.6)	1.03 (1.08)	0.71 (0)	1.01 (0.16)

* significant differences by language. For sample sizes greater than 300, depend on the histograms and the absolute values of skewness and kurtosis without considering z-values. Either an absolute skew value larger than 2 or an absolute kurtosis (proper) larger than 7 may be used as reference values for determining substantial non-normality.

Response Options: 0 = never, 1 = rarely, 2 = sometimes, 3 = often, 4 = always; higher mean subscale scores correspond to greater social support for that behavior from that source (family, health providers, or others/friends).

#### Internal consistency.

The final OHBSS scales exhibited adequate internal consistency, reflected by robust Cronbach’s alphas and McDonald’s omegas (which were near identical), in both languages all ≥ 0.953 (lower CI bound ≥ 0.944); English all ≥ 0.954 (lower CI bound ≥ 0.944), and Spanish all ≥ 0.946 (lower CI bound ≥ 0.926) (Table 15). These scores provide evidence in support of the final scales’ reliability, in the full sample, and for both language groups.

**Table 15:** Internal consistency for final OHBSS scales, overall and by language.

	Cronbach’s Alpha (95% Confidence Interval)			McDonald’s Omega (95% Confidence Interval)		
Full Sample (n = 502)	Family	Health Providers	Others/Friends	Family	Health Providers	Others/Friends
Brushing	0.957 (0.951, 0.962)	0.953 (0.944, 0.960)	0.968 (0.962, 0.974)	0.957 (0.951, 0.962)	0.953 (0.944, 0.960)	0.968 (0.963, 0.974)
Flossing	0.970 (0.966, 0.973)	0.962 (0.955, 0.967)	0.979 (0.973, 0.983)	0.971 (0.967, 0.974)	0.962 (0.955, 0.968)	0.979 (0.973, 0.983)
Dental Care	0.961 (0.955, 0.966)	0.958 (0.951, 0.964)	0.969 (0.963, 0.975)	0.961 (0.956, 0.966)	0.957 (0.950, 0.964)	0.969 (0.963, 0.975)
**English (n = 303)**						
Brushing	0.955 (0.947, 0.962)	0.956 (0.946, 0.964)	0.971 (0.963, 0.977)	0.955 (0.947, 0.962)	0.956 (0.946, 0.964)	0.971 (0.963, 0.977)
Flossing	0.968 (0.962, 0.974)	0.963 (0.954, 0.969)	0.978 (0.971, 0.983)	0.969 (0.963, 0.974)	0.963 (0.954, 0.969)	0.979 (0.971, 0.983)
Dental Care	0.958 (0.950, 0.965)	0.954 (0.945, 0.964)	0.969 (0.961, 0.976)	0.958 (0.950, 0.965)	0.954 (0.944, 0.963)	0.969 (0.960, 0.976)
**Spanish (n = 199)**						
Brushing	0.957 (0.945, 0.967)	0.946 (0.926, 0.960)	0.963 (0.951, 0.972)	0.958 (0.945, 0.967)	0.946 (0.926, 0.960)	0.964 (0.953, 0.973)
Flossing	0.971 (0.964, 0.976)	0.961 (0.950, 0.970)	0.979 (0.971, 0.985)	0.973 (0.966, 0.978)	0.962 (0.951, 0.970)	0.979 (0.972, 0.985)
Dental Care	0.966 (0.957, 0.973)	0.963 (0.950, 0.973)	0.969 (0.959, 0.977)	0.966 (0.957, 0.974)	0.963 (0.949, 0.973)	0.968 (0.958, 0.977)

## Discussion

The dual-focus co-creation approach (i.e., creating two language versions simultaneously) was successful, and the final OHBSS scales in English and Spanish are comprehensive in terms of coverage of key structural and functional dimensions of social support for three important oral health behaviors. The new scales also assess social support from different sources, which has implications for use in future behavioral intervention research and targeting different potential sources of social support. Our approach utilized independent samples for each study, and ensured scale development followed evidence-based guidelines for achieving conceptual, content, and normative equivalence in both languages. Co-creating new scales in two languages simultaneously for behavioral research is relatively unique. The dual-focus approach is feasible, with proper planning and support, and promotes equity. Items were generated bi-directionally, sometimes in Spanish first, sometimes in English first, with items written from the source interviews in Study 1. Beginning with an exploratory qualitative phase and literature review we created a large item bank in both English and Spanish, which is an ideal starting point for scale development.

Some types of social support were easier to capture than others and may be most relevant for our target dental behaviors; this can be observed in the greater number of items on informational and instrumental types of social support, and fewer emotional or appraisal support items. It is possible the latter types of support (e.g., emotional) may not be as relevant for the widely applicable behaviors of toothbrushing, flossing, and dental care, unless individuals have strong dental phobias. The fact that there were differences in OHBSS scale scores across sources of social support was expected, suggesting potential avenues for intervention and the scales’ sensitivity to detect changes in social support from these different sources. A potential application is the ability to compare differences in sources of support and identify where more support may be offered. While the “Others/Friends” group was often an infrequent source of social support, CHWs are part of this group, and they may be trained to promote oral health in various aspects. CHWs have provided oral health education and supported changing behaviors and supporting access to dental services [91].

The optional items also appear to be valuable screening tools and identified additional needs that were applicable to many, but not all. The screening questions in the optional section are brief and have important implications for utility in clinic settings, especially among FQHCs that serve many lower-income populations who struggle with these barriers to care.

The longest existing social support scales have 40 items, according to a 2011 review by Lopez and Cooper [30]. Thus, the 37-required unique item count (and 44-items, if all optional items are included) in the OHBSS scales are not excessively long. The OHBSS scales offer flexibility in assessing social support from three different sources. Structuring the scale in a way to allow rating one item three times (once for each source of support) is efficient and minimizes the respondent burden. Shorter social support scales are less likely to capture multiple dimensions [31], with a potential tradeoff between length and utility.

The final OHBSS scales exhibited structural validity and internal consistency overall, and in both English and Spanish. Very minor revisions were required when exploring model fit indices. There were more English-speakers than Spanish-speakers in Study 3, though there did not appear to be substantial differences by preferred language. Although there were more English-speakers, it is notable that many participants were bilingual, and had the choice of which language they preferred to complete the survey. Future analyses will explore other psychometric properties of the final scales, overall and in each language.

While we focused on one priority population of 21– to 40-year-old Mexican-origin men and women to develop the scales, future work can expand upon this demographic, and test the scales further for generalizability to other Hispanic/Latino heritage groups. There may also be differences among Hispanic/Latino heritage groups living further from the US-MX border or in other geographic regions outside of the Southwest. Our priority population for scale development primarily captured one cohort, millennials (also called “Gen Y”), born between 1981-1996 [92]. In addition to context of the recent global COVID-19 pandemic, there may be other important or unique age-period-cohort effects experienced by our study samples that could have affected our results and final scales. Future multivariable modeling and analyses can account for other sample characteristics.

### Strengths and Limitations

Significant study strengths include our rigorous mixed methods and bilingual co-creation approach. We collected rich life course perspectives in our qualitative Study 1, which had a large sample size for qualitative inquiry; these data will be explored in more depth in future analyses. Our dual-focus approach and detailed documentation of our translation and bilingual item bank co-creation steps are a major study strength that can serve as a template for other scale development efforts. Adjudicating the interview guide was a time-intensive step in the formative phase, but was critical and shaped how we asked about the constructs of interest, which informed later phases of scale development. OHBSS scales were co-created in two languages simultaneously, which is not frequently done or described in scale development, and is the first to our knowledge to adopt this approach for an oral health behavior scale. This parallel processing approach appeared superior to other translation methodologies like back-translation or serial translation, and kept one language from dominating the other. While it was challenging to coordinate a large multi-site study team and expert panel for all study phases, we succeeded in bringing together the necessary expertise, including input from community members and clinic partners throughout. We built on a long history of strong collaboration with community partners, which was critical for successful outreach to our priority demographic group. Trust, flexibility, and regular updates and communication (standing meetings with each clinic partner at least once/month) enabled our teams to shift and adapt as needed during the pandemic to still meet study goals.

We achieved successfully balanced samples by languages across all three studies; samples were well-balanced by marital status, and to a lesser degree by sex. Recruiting and enrolling men was very challenging in all three studies, even with spouses/partners and other family members living in the same household being eligible. This is not uncommon in previous research. A study sample of at least 30%-50% men was needed to power meaningful sex-stratified statistical analysis. The rationale for striving for balance across these characteristics was due to documented differences in the sources and types of social support for men and women [31,93]. Notable sex differences have been observed in racially/ethnically diverse adults, with women engaging in more frequent oral hygiene behaviors than men and visiting the dentist more often [94–96].

Data collection for Studies 2 and 3 occurred during the global COVID-19 pandemic, which necessitated shifting to remote outreach, recruitment, and data collection. Conducting online surveys may have introduced some biases in our sampling in favor of respondents who were already more familiar with and had better access to internet/ e-media technology. Study staff offered alternative options for survey completion including having an interviewer administer the entire survey via phone, or mailing a hard copy, but respondents did not request these modalities. Study staff were able to assist participants with the screening and enrollment process via phone call and texts, and in some cases stayed on the phone ensuring participants could access the unique survey links and start the survey or continue where they left off.

### Conclusion

This study yielded new, culturally-relevant, oral health behavior-specific social support scales in English and Spanish for oral hygiene behaviors (brushing, flossing) and dental care utilization from family, health providers, and others/friends. The final OHBSS scales exhibited structural validity and internal consistency, overall and by language.

## Supporting information

S1 Table Study 1 interview guides in English and Spanish. (PDF)

S2 Table Study 1 codebooks. (PDF)

S3 Table Study 2 EFA item loadings. (XLSX)

S4 Table OHBSSv3 scale. (PDF)

S5 Table Final OHBSS scales. (PDF)

S6 Table Final OHBSS items. (PDF)

S7 Table Final OHBSS item descriptive frequencies. (PDF)
