# Supplementary material for: Development of new bilingual oral health behavior social support (OHBSS) scales in English and Spanish
Source: PLoS One. 2025 Mar 11;20(3):e0317133. doi: 10.1371/journal.pone.0317133 (PMC11896079; doi:10.1371/journal.pone.0317133)
Supplement: S2 Table — (PDF) [file pone.0317133.s002.pdf]

S2. OHBSS Study 1 Codebook

AIMS:

Primary: Qualitatively explore the various types and sources of social support on oral hygiene behaviors and dental care utilization among lower-income Mexican-origin young adult men and women.

Secondary: Identify other sources of influence (social, sociocultural, etc.), both positive and negative, on oral hygiene behaviors and dental care utilization.

GOALS:

Use qualitative data to develop a quantitative self-report scale that captures types and sources of social support for oral hygiene behaviors (brushing, flossing, rinsing) and dental care utilization that may be modifiable in a future intervention study.

- Proposed draft OHBSS length: ~ 40 items
- Proposed final OHBSS length: ~20-24 items

METHODS:

Semi-structured interviews: 72 participants

Target population: predominantly lower-income Mexican-origin young adult men and women (ages 21-40 years old), single or married/living as married

Balanced recruitment: sex, marital status and language

Language: English or Spanish

Target behaviors of interest: Oral hygiene behaviors and dental care utilization

Sites: North San Diego County (SD) and Imperial County (IC), CA

DIMENSIONS OF SUPPORT:

Items will represent approximately four constructs/themes:

Informational support represents a third type of social support (one that is sometimes included within the instrumental support category) and refers to the help that others may offer through the provision of information.[1] Advice, suggestions, and information[2]

Instrumental support refers to the various types of tangible help that others may provide (e.g., help with childcare/housekeeping, provision of transportation or money).[1] Tangible aid and service[2]

Emotional support refers to the things that people do that make us feel loved and cared for, that bolster our sense of self-worth (e.g., talking over a problem, providing encouragement/positive feedback); such support frequently takes the form of non-tangible types of assistance.[1] Expressions of empathy, love, trust and caring[2]

Appraisal support refers to information that is useful for self-evaluation[2]

Items will consider different target oral hygiene behaviors - brushing, flossing, and rinsing.

Items will consider different sources of support including family and friends.

RESPONSE OPTIONS:

Frequency of received support (over the past week) is a potentially effective approach for a future intervention study.

SUPPORTING THEORIES:

This scale development study is guided by a modified version of the Lee and Divaris (2014) unified oral health disparities framework[3], which includes social support as factor influencing oral health-related behaviors and oral health outcomes. We also referenced the MacArthur Research Network[1], health behavior theories[2], our

behavioral model for vulnerable populations adapted for Mexican migrants and dental care,[4] social support theories[5], and the existing literature about the types and sources and possible roles of social support in health[6].

References

1. MacArthur Research Network on SES and health: https://macses.ucsf.edu/research/psychosocial/socsupp.php#definition
2. Glanz, K., Rimer, B. K., & Viswanath, K. (Eds.). (2008). *Health behavior and health education: Theory, research, and practice* (4th ed.). Jossey-Bass. http://www.med.upenn.edu/hbhe4/part3-ch9-key-constructs-social-support.shtml
3. Lee and Divaris (2014). The ethical imperative of addressing oral health disparities: A unifying framework. J Dent Res 93(3): 224-230.
4. Velez, D., et al. (2017). Facilitators and Barriers to Dental Care Among Mexican Migrant Women and Their Families in North San Diego County. J Immigr Minor Health. 19(5): 1216-1226.
5. Cohen, S., et al. (2010). Social relationships and health. Social support measurement and intervention: A guide for health and social scientists. S. Cohen, Gottlieb, B., Underwood, L.G. New York, NY, Oxford University Press.
6. Lopez, M. L. and L. Cooper (2011). Social support measures review-Final Report, National Center for Latino Child and Family Research

CODEBOOK 1			
**Domains** (in grayed rows)			
**Constructs**	**Definition**	**What it is not**	**Sample quote**
Oral hygiene behaviors			
Brushing	Here we just capture references to the actual behavior of brushing teeth.		*I brush my teeth in the morning and again at night.*
Flossing	Here we just capture references to the actual behavior of flossing teeth and other interdental cleaning.		*And in the morning, I floss with the swordy thing because I don't like to floss.*
Rinsing	Here we just capture references to the actual behavior of rinsing mouth, including rinsing with mouthwash or just water.		
Other OH behaviors	This can include but is not limited to tongue cleaning, wiping teeth.		*I wipe my teeth with parts of a burned tortilla to my clean teeth.*
Product	Mention of the specific product used in any of the OH behaviors (e.g., toothpaste type, brand, interdental cleaner type). This includes description of product, and is not limited to brand name.		*I use the soft, flexible plastic pick to get in between my back teeth. I can't reach it or clean it well otherwise.*
Foods and beverages	Connections between food and/or beverages and oral health (including dental hygiene behaviors); avoiding certain foods that are bad for oral health or seeking out food that is good for oral health.<br><br>This includes food/beverages that impact oral health behaviors.		*When I eat meat it gets stuck in between my teeth so I use floss.*<br><br>*I avoid eating candy to not get cavities.*
Initiation of OH behavior - PAST	How the individual first learned or started the OH behavior; how a routine was developed. This will be co-coded with behavior and time periods.		*I learned how to floss from my mom.*
OH behavior routine – CURRENT	When and where the individual engages in current OH behavior and how. This will be co-coded with behavior and time periods. Here are possible behavioral dimensions:<br>● Frequency<br>● Duration<br>● Use of reminders/prompts<br>● Location		*I brush my teeth twice a day and use mouthwash 3 times a day.*<br><br>*I brush my teeth for 2 minutes twice a day.*
Changes in the OH behavior routine	Change in their routines in how, when, and where they practice their OH behaviors. This will be co-coded with behavior and time periods.		*When I moved to the U.S. I started flossing twice a day instead of once.*

Occasional OH behavior	Instances of periodic or unusual oral hygiene behaviors, will include the examples people give of what they do if they skip their normal routine		*When I stay over at my partner's house I do not use mouthwash.*
Care access and utilization – both dental and healthcare			
Health care	Professional medical care. Provided by a licensed health professional (physician, nurse, etc)	Not professional dental care.	
Dental Home	Refers to having a usual source of dental care		
Orthodontics	References to the implications of wearing or having worn/wearing braces or clear aligners. This includes dental visits for braces and/or other types of orthodontic treatment (includes references to expanders, retainers, headgear, etc).<br><br>This may get co-coded with an oral hygiene behavior whose practice is affected by the braces or other dental treatment.		
Other dental treatment	References to other non-preventive dental care treatments (oral surgeries, implants, extractions, fillings, crowns, wisdom teeth, etc)		
Preventive dental care	Professional dental care, which can include a range of preventive dental care/ treatments/services/visits. This includes checkups, cleanings, general comprehensive oral examinations, fluoride varnish, sealants, and other types of preventative care. This care can be provided from different types of licensed dental providers (dentists, hygienists)	Not dental treatments (fillings, etc)	
Dental care pattern	Reflects discussion of frequency/timing of dental visits; regular attender		*I go to the dentist every six months.*
Medications	Need for medication before, during, or after dental visit in order for care to be provided.<br>Would include any type of medication (examples might include anti-anxiety medications; pain management; sedation etc)		
COMMON CODES			
Good Quote	A quote that captures a concept really well and would be a good example to share at a presentation or in an article, and/or is unusual		

Facilitators/ positive valence	References people, opportunities, resources, or anything that makes it more likely to engage in the target OH behaviors in this section (hygiene or dental service use) ;<br>Helpful, supportive or reinforcing		
Barriers/ negative valence	References challenges, obstacles, or anything, including people, that make it less likely to engage in the target OH behaviors in this section (hygiene or dental service use); NOT helpful		*I don't have enough money to buy mouthwash.*
REVIEW	This needs closer review. It may be an something unusual or a possible emerging theme that needs a new code and does not fit in the existing codes		

**CODEBOOK 2**			
**Domains** (in grayed rows)			
**Constructs**	**Definition**	**What it is not**	**Sample quote**
People – interactions with individuals or groups			
Spouse/ Partner	Individual the respondent identifies as non-biologically related social network member with whom he/she has chosen to share life with; usually involves the sharing of resources and planning the future together. Relationship can be current or former	Not a friend with a close, long-term relationship (List under Friends)	
Children	Children of the interviewee, including biological, step, foster, adopted etc. Include children who may not be yours but that you have raised as your own		
Parents	Individuals the respondent identifies as a mother or father, including biological, step, adoptive, and foster parents	Not a family caregiver such as a grandmother (list under Extended family members)	
Other Immediate family members	Immediate family members including siblings, step-siblings, and others raised as close family members	Not children or parents	
Extended family members	Extended family members including grandparents, aunts, uncles, cousins, godparents, in-laws, nieces/nephews		
Friends	Non-familial social network members including neighbors from whom the individual seeks and/or provides support and assistance; includes individuals who are not biological or step-family members but are referred to as an aunt/cousin, etc.	Relatives	*A friend of mine, well she's my child's godmother…*<br><br>*My aunt, well she's my mom's friend...*
Coworkers	Nonfamilial social network members with whom the individual works and from whom the individual seeks and/or provides support and assistance		*Well sometimes I ask someone at work to take me to my dentist appt. if I don't think I can there in time.*
Dental care pros	Individuals from whom the individual seeks and/or receives dental care, including but not limited to dentist, dental educator, auxiliary staff (e.g., receptionist, dental assistant)	Not medical professionals	*Yes, he (dentist) got angry with me because I was not flossing like he told me a should.*
Health care pros	Individuals from whom the individual seeks and/or receives medical care, including doctors, nurses, health educator, community health workers, and auxiliary staff (e.g., receptionist, medical assistant)	Not dental care professionals	*Well, because of my weight, my doctor said I need to change my diet. Maybe if I stopped drinking so much soda, this might be good for my teeth too, right?*

Other organizational members	Individuals such as teachers, coaches, pastors or priests, social workers, WIC		
Other people	Individuals not captured by rest of the people codes, this can include roommates; this may include other patients of a shared dental/health care organization		
Organizations – interactions with various types of organizations; global institution-level			
Healthcare Organizations	Healthcare organizations including but not limited to private dental offices, clinics (e.g., CDSDP), hospitals, emergency rooms, includes mobile dental units		*At CDSDP health education workshop, I obtained information on proper flossing techniques.*
Church	Religious organizations		*At my church I received free toothbrushes.*
School	Sources that are described at the organizational level related to educational institutions, including the respondent's own as a child, and as a parent whose child is in school. This includes all types of academic institutions from kindergarten to 4-year universities.<br><br>In Imperial County, this can include IVROP.		*My son's school sometimes sends flyers home about health issues. I remember one flyer that was about where to get free dental care.*
Work	Sources that are described at the organizational level, including at a caregivers' place of employment		*I went to my work's wellness workshop Lunch and Learn on oral hygiene and got a free toothbrush.*
Community-based	Government and non-government organizations (e.g., WIC, other charity organization sponsored health fairs, etc)	Not healthcare specific	*I only get dental care when the mobile unit that offers free care is in town and hosts a health fair event in my neighborhood.*
Other organizations	Other organizations not captured by the above codes		
Communication channels/modes/types, including electronic, interpersonal, print			
Internet	Sources found on the internet/websites, this includes information received via subscribing to automated updates from a site or source.	Not social media	*I Googled it. I asked Siri. I looked it up on WebMD website. I watched a video online on youtube about how to floss with braces.*
Mass media	Television, radio, billboards, newspapers including community newspapers (e.g., Pennysaver)	Not include print material	*I saw a TV commercial for a fancy new toothpaste with small listerine strips in it and I wanted to try it out*
Social media	Sources such as Facebook, Twitter, Instagram (interactive sites online). This would include posting in a	Not a back and forth interaction (see interpersonal)	*I saw a Facebook event page about a dental health fair*

	public/general sphere that includes people PID does not know.		*I posted on my FB page to get recommendations for a new dentist.*
Mobile applications	Health-related apps (like Text2Floss, Kaiser app). Anything that lives inside the app (info; videos; reminders)	Not social media; not links to external websites	*My Kaiserapp notified me that I was due for my next appointment.*
Interpersonal	Directly communicating with a real person by any means/mode (in person, text, phone, or e-mail). This includes mobile app-supported platforms for text/video like WhatsApp, WeChat, Skype). Could be reciprocal, but does not have to be back-and-forth discussion. PID knows the person/people communicating with. Includes reminders from dentist.	Not an automated text or email generated from other sources	*My husband texted me a reminder this morning that I had a dental appointment this afternoon.*<br><br>*It was my sister who told me that I should change dentist.*
Print material	Print materials like flyers, handouts, brochures or pamphlets		*I received a booklet about managing oral health and diabetes from the promotora.*<br><br>*I picked up a flyer about dentical that I saw at a booth at the fair.*
Classes	Structured activity involving a group of people, usually in person and delivered in a workshop-type settings		*At CDSDP health education workshop, I obtained information on proper flossing techniques.*
Other channels/modes/ types	Other communication channels/modes/types not captured in codes above		*The label of the mouthwash gave me information on how to use.*
Contextual factors			
Childhood	This period of development includes when the interviewee was a child, age 11 and under, and experiences that occurred then, if it is not clear that PID was an adolescent, code it as childhood.	Not the PID's child.	*Growing up I was a Colgate kid.*
Adolescence	This period of development includes when the interviewee was a child between age 12-17, and experiences that occurred then, this includes reference to being a teen/teenager.	Not the PID's child.	*When I was a teen I got braces.*
Young adulthood	This period of development includes when the interviewee was a young adult, between age 18 and 26 (age when off parent's insurance).<br>This includes "leaving the nest", apply this code if PID is under age 27 at the time of interview.		*It was really helpful to stay on my parent's health insurance plan in my early 20's before I found my first job with benefits.*

Adulthood	This period of development includes experiences that occurred when the interviewee was an adult, age 27 and above. If it is not clear what age PID was when referencing historical experience then code adulthood if PID is aged 27 or older at the time of interview.		*Last year I chipped my tooth and had to get it extracted.*
Parenthood	Things occurring or learned through interviewee's role as a parent		*I took my daughter to the dentist and the dentist taught me a few things on how to care for my own oral health.*
Country	Specific references to any activity occurring in a country other than the US.		*I went to Mexicali to get my teeth cleaned.*
Living situation	Life context more generally, including independent living situation versus not; sharing bathroom; can include both past and present living situation		*My kids walk into the bathroom when I am brushing my teeth.*<br><br>*In college I had a roommate who just rinsed her mouth with water.*
Other contextual factors	Other contextual factors not captured by the above codes		*In the rehab center where I lived I learned the importance of oral health.*<br><br>*The local store runs out of my type of toothpaste.*
COMMON CODES			
Good Quote	A quote that captures a concept really well and would be a good example to share at a presentation or in an article, and/or is unusual		
Facilitators/ positive valence	References opportunities or resources or anything related to a person or place or contextual factor that makes it more likely to engage in the target OH behaviors;<br>Helpful, supportive or reinforcing		
Barriers/ negative valence	References challenges, obstacles or anything related to a person or place or contextual factor that makes it less likely to engage in the target OH behaviors ; NOT helpful; laziness is considered a barrier		
REVIEW	This needs closer review. It may be something unusual or a possible emerging theme that needs a new code and does not fit in the existing codes. Include a memo with a brief note about what needs to be reviewed/discussed. Include a memo		

	with a brief note about what needs to be reviewed/discussed		

**Codebook 3**			
Possible types of support and sources of influence (positive and negative). All support is two-way / interactive and can be received or provided (co-code with support given). These will be co-coded with positive or negative valence. Facilitators are when these factors are supportive; barriers are when the factors do not facilitate OH behaviors and/or dental care utilization.			
**Constructs**	**Definition**	**What it is not**	**Sample quote**
Types of support - what are people doing that is support, directly or indirectly - also includes references to 'lack' of a type of support (co-coded with valence)			
Information	Advice, suggestions, guidance from others to promote the behaviors; this includes activities that may promote knowledge acquisition.<br><br>Statement that someone gives a recommendation or provides guidance/advice on anything related to oral health. This also includes the PID giving reminders to others (co-code with support given)<br><br>They do not necessarily need to follow through with the advice.	Do not include skill acquisition (teaching them how to do something); this goes under Teaching/skill development	*At a health fair a hygienist taught me the importance of flossing and suggested I do it every night because it helps keep the space between my teeth clean.*<br><br>*My coworkers recommended a dentist to me, so I looked him up and I have been going to him for 2 years now.*
Lacking Example:<br>Nobody referred me to my dentist I found him myself online.			
Reminders	Statement that someone/something (e.g. call/text/email from dentist) reminds the PID to engage in an oral health behavior.<br><br>This also includes the PID giving reminders to others (co-code with support given)<br><br>This will be co-coded with the source of the reminder.	Do not include attempts to educate or teach.	*My sister reminds me to brush my teeth every night when she gets up to brush her teeth. So then when I see her doing it, it reminds me that I need to brush my teeth too.*<br><br>*My husband reminded me that we needed mouthwash.*<br><br>I tell my son to brush his teeth all the time…<br><br>My dentist sends me a reminder when it is time for my checkup
Lacking Example:<br>The staff at the dental clinic would not provide me with reminders which I would have found helpful.			
Emotional	Mentioning of support in the form of praise, warmth, caring, showing love and/or affection etc. This can be provided to the PID.<br>This also includes the PID giving this type of support to others (co-code with support given)		*My dentist told me I was doing a good job brushing in circles so everytime I brush my teeth I continue to brush in circles.*

			I cheer on my son when he flosses
Lacking Example:<br>My mom used to encourage us to brush our teeth, after my dad passed away she doesn't provide that to us anymore.			
Instrumental	Active aid and assistance provided to the individual in order to support the behavior.<br>This also includes the PID giving aid to others (co-code with support given)<br><br>This can come in many forms, such as: a ride or transportation to the dentist, providing childcare to facilitate going to a dental appointment; purchasing dental hygiene products and tools, and making them available; recommending a dental clinic that accepts their insurance; calling to schedule an appointment.<br><br>This will get co-coded with sources of influence below.	Not information, emotional or financial support	…. and [I] pack a toothbrush and toothpaste in his lunch box.<br><br>I gave my mom some new mouthrinse to try that I got.<br><br>My aunt babysits for me if I have doctor or dentist appointments in the afternoons.<br><br>The clinic care coordinator called me to make sure I got my new Medicaid card, and to let me know I am covered for dental now and to see if I needed help to make an appointment.
Lacking Example:<br>I don't really have anyone to drive me to the dentist, and I don't know how to drive, so I end up not going.			
Teaching/Skills Development	Mention of receiving teaching/instruction on how to engage in an OH behavior, or how to find/access/fully utilize dental care.<br><br>Building behavioral capacity through direct instruction; this can include providing feedback (appraisal support).	Not passive learning by reading.<br><br>Not passive learning by observing someone engaging in the behavior.	*Now I know how to brush my teeth properly because they taught me how to do it in school.*
Lacking Example:<br>My mom never actually taught me how to use mouthwash I just learned from watching her do it.			
Observational learning/Role modeling	Mention of seeing someone engaging in a target behavior, and mention of engaging in a learned behavior through observing somebody else.<br><br>This also includes the PID describing serving as a role model for one of the target behaviors for others.(co-code with support given)	Not direct teaching.	*I saw my boyfriend flossing and I started to floss only that I use the picks and not the floss because I don't like using floss.*<br><br>*My son learned how to floss by watching me do it.*
Other support	Other ways in which someone (or a group of people) have helped the PID.		

	Other ways PID provides support - types not listed above (co-code with support given)		
Sources of influence - what factors, positive or negative, including but not limited to motivations, that are influencing the OH behaviors and dental care use; these are now organized from individual factors (e.g, what a person might feel, think, etc) to 'higher' levels of influence such as social norms (e.g., what my community thinks about a behavior). - includes references to 'lack' of a source of influence (co-coded with valence)			
Intrinsic motivation	Factors internal to the individual that influence engagement in an oral hygiene behavior and/or dental care use.<br><br>This includes the individual initiating a behavior themselves.	Do not confuse this with health beliefs or other sources of influence like social norms/social pressures.	*I bring myself to brush my teeth despite my laziness because I like the feeling of freshness afterwards.*<br><br>*Since I have coffee stains on my teeth I wanted to start using mouthwash for this so I researched the best one for me and read the instructions and now I use it everyday.*
Lacking Example:<br>I feel really lazy to floss every night so I skip it.			
Sensations	References to sensations experienced in the mouth, including fresh or bad breath (co-code with positive or negative valence), burning or mouthwash, "yucky feeling" if you don't brush, such as food getting stuck with braces.<br><br>This may be co-coded with food/beverage.	Not pain from caries or periodontal disease	*I like feeling the burning in my mouth when I rinse because then it feels like I am really getting my mouth cleaned.*<br><br>*My teeth feel so nice and smooth after I clean them.*<br><br>*I don't like having bad breath.*<br><br>*I feel good after I am done brushing my teeth because I feel like I did something great for my health.*
Lacking Example:<br>I don't really care for fresh breath so I don't use mouthwash.			
Dental pain	Pain in teeth or mouth to include pain when engaging in oral health behavior as well.<br><br>This can include pain due to caries or periodontal disease (e.g., sensitivity to cold, pain when chewing), dental procedures/treatment (e.g., braces), or dental hygiene behaviors (e.g, flossing).		*My teeth really hurt when I floss them so I try not to floss too often.*<br><br>*I started to notice that biting ice was hurting my teeth so I stopped biting the ice in my drinks.*
Lacking Example:<br>I have never had any pain in my teeth so I have never gone to the dentist since my teeth are fine.			

Dental fear/anxiety	Procedure related, as well as general experience with anxiety or fear related to oral hygiene behaviors and dental care, including but not limited to the noise or the instruments		*Sometimes I get nervous going to the dentist because of the noise the little machines make they give me the creeps sometimes.*
Health	Comments related to one's own health condition that may impact decisions/behaviors; needing to modify behaviors given other diagnosed health condition such as diabetes	Not beliefs about the consequences of poor OH behaviors or lack of dental care	*I have diabetes so I limit the amount of sugar I consume.*<br><br>*I take some meds that create more dry mouth*<br><br>*I have a heart condition that requires pre-medication before a dental procedure*
Health beliefs	Thoughts or perceptions related to the link between OH behaviors and dental care use and the immediate and longer term consequences on health outcomes / quality of life, including beliefs about reasons for seeking care/engaging in an oral health behavior.<br><br>This also includes comments related to perceptions of control over one's health/oral health behaviors.<br><br>Self appraisal of need for engaging in the oral health behavior.<br><br>Reasons stated as important to seek dental support.		*I stopped flossing because I got an infection and my dentist told me to stop.*<br><br>*When I walk barefoot on the floor I get cold sores.*<br><br>*It doesn't really matter what I do about my health.*<br><br>*Nothing is bothering me right now so I don't go to the dentist.*<br><br>*I don't think I know how to brush my teeth correctly.*
Spirituality	This relates to beliefs, practices or references to God, higher others, church, etc. and engagement in religion		*I pray to God when I need help with anything. I prayed for my toothache to be gone in the morning after I took the aspirin at bedtime the night before*
Language	This references language playing a role in dental care/oral health (co coded with barriers or facilitators)		*I struggle to understand what my dentist is saying. He tries to speak Spanish but does not really know how.*
Time	This refers to the amount of time related to engaging in oral health behaviors.This includes discussion of lack of time or other time considerations, such as length of time for scheduling a dental visit, wait time		*Sometimes in the morning, I wake up too late and don't have time to floss.*<br><br>*I hate going to the dentist because no matter what*

	at the dental office before being seen, dental office hours, (structural aspects of provider availability; walk-ins; offer evening hours), making time for taking care of own oral health needs (like brushing in the morning)		*time they schedule me, they always make me wait hours before my scheduled appointment time.*
Transportation	Anything related to transportation and oral health, such as providing a ride to the dental office, loaning a car, paying for gas or bus/trolley token, not able to get to the clinic (too far; no car; no bus line)<br><br>Co-code with “instrumental” and source of support.<br>Co-code with source given if PID provides this type of support.		*If we have just one working car, then I work with my husband’s schedule to get a ride to the dentist.*
Financial	Monetary assistance/support, including but not limited to covering the cost of dental care visits, oral hygiene products, gas or a ride (co-code with transportation), staying on parent or spouse’s dental health insurance (co-code with insurance)<br><br>Can include any discussion of financial strategy (e.g., buying toothbrushes on sale)<br><br>Co-code with “instrumental” and source of support.		*I didn’t have an idea about how much it was. My parents paid for it and if they didn’t, then we wouldn’t have it [dental care]*
Insurance	This includes any reference to health insurance coverage, including dental (signing up for insurance, scope of benefits, etc)<br><br>Co-code with “instrumental” and source of support.		*I don’t have dental insurance, so I only go to the dentist in Mexicali if I need anything, not to one here on this side [in US].*<br><br>*I had a question about my insurance, I wasn’t sure if Medi-Cal covered dental insurance.*
Childcare	Anything related to childcare and oral health. This can include tangible assistance with babysitting children to enable parent to seek dental services or take care of own oral health needs; or not going to the dentist since need to care for children.<br><br>Co-code with “instrumental” and source of support.		*Difficulties may be like working around my husband’s work schedule and doing appointments whenever he’s not at work so he can take care of our son.*

Lacking Example:<br>I have nobody who can help me take care of my children so it makes it tougher for me to go to my dental check-ups.			
Social norms/social pressures	What individual states/believes is accepted as 'standard practice' for the referred group. Norms may be derived based on cultural or contextual influences, for example, and may eventually influence what a person does. Includes unspoken rules, what is considered common courtesy, socially acceptable		*It is just common courtesy to not talk to someone up close when you haven't brushed your teeth.*<br><br>*It is typical in my family not to floss. We are just not used to doing it.*<br><br>*I know everyone is supposed to brush 2-3 times a day.*
Lacking Example:<br>In my house we didn't really use mouthwash except very rarely but we were never used to it so I didn't think that it was something that I needed to do.			
Trustworthiness	This relates to how valid/correct the information/advice or reliable a person is perceived to be when offering aid/advice/support.		*My friend said I should check how that dentist rated on Yelp, and I think the ratings on that website are usually pretty true and that's how I picked my doctor.*
Lacking Example:<br>I don't really trust the websites with the list of dentists I would rather go to a dentist that someone I know recommends to me.			
Punitive/ Punishing	Mention of receiving shaming, blaming, put-downs, nagging etc. for a presence or lack of an oral health behavior.		*My husband becomes really upset with me if I do not brush my teeth at night and he nags me, so I brush my teeth to avoid his nagging.*
Precipitating event	An occurrence that results in new actions taken; this can include but is not limited to a diagnosis of a health condition/death in the individual or family member, migration to the US (or in-migration within home country).		*When my dad passed away my mom became very controlling of our time and did not let us brush our teeth because she wanted us with her at all times.*<br><br>*When I started dating my girlfriend I started focusing more on my dental health because of her oral hygiene practices.*
Other influences	This code should be used to capture any other source of influence on an		*I floss twice a week really even though I would want to do it more often I just*

	individual's behavior, including both internal or external influences.<br><br>References to the difference between what an individual plans/planned to do versus what they actually do/did.<br><br>This can include perceptions about the value of something.		*don't like the feeling of flossing because my teeth are too close together.*<br><br>*I never rinsed before because I never really felt like I needed to.*
Support Given	Apply this code as a co-code when PID references providing support to others in any way.<br><br>This will be co-coded with others		*I always tell my son that he needs to brush his teeth at school even if he is embarrassed because of his braces and I pack a toothbrush and toothpaste in his backpack.*
COMMON CODES			
Good quote	A quote that captures a concept really well and would be a good example to share at a presentation or in an article, and/or is unusual		
Facilitators/ positive valence	Opportunities, resources that make it more likely to engage in the target behaviors, for example, resources for feedback and instruction on how to floss. Something or someone that/who is helpful, supportive or reinforcing		*I am really glad I found this dentist.*<br><br>*I have heard of another dentist that is really good and has helped many people but I am comfortable with my dentist.*<br><br>*At my niece's event in school they gave plenty of useful information on oral health. I learned a few things from that event.*
Barriers/ negative valence	Challenges, obstacles that make it less likely to engage in the target behaviors, for example, lack of resources/instruction on how to floss; NOT helpful.		
REVIEW	This needs closer review. It may be an something unusual or a possible emerging theme that needs a new code and does not fit in the existing codes. Include a memo with a brief note about what needs to be reviewed/discussed		
