# Supplementary material for: Development of new bilingual oral health behavior social support (OHBSS) scales in English and Spanish
Source: PLoS One. 2025 Mar 11;20(3):e0317133. doi: 10.1371/journal.pone.0317133 (PMC11896079; doi:10.1371/journal.pone.0317133)
Supplement: S4 Table — (PDF) [file pone.0317133.s004.pdf]

Dental Health Support Survey – OHBSS Study 3, v3.17.2022

BOCA SANA CUERPO SANO

INSTRUCTIONS:

This survey asks about help or social support **you might get from 3 different groups of other people** for different dental health behaviors. Please rate each statement **3 times** - once for each of these groups of people:

1) **Family**: includes your significant other, and any immediate and extended family members you may or may not live with
2) **Health providers**: includes medical providers, dentists and dental specialists
3) **Friends and other people**: includes friends, neighbors, co-workers etc.

Please think about and answer questions based on your current dental experiences and behaviors you do now, as an adult. There are no right or wrong answers. We are interested in who supports you now. Please select the best answers for each question, and do not leave anything blank.

Please look at the example below. Remember to rate each statement 3 times (from left to right →), and make sure each group of people has a rating. If the statement does not apply to any of the groups for you, then please check "Never."

**For each statement, how often do you get support from each group of people?**	**1. FAMILY** (includes your significant other, and any immediate and extended family members you may or may not live with)					**2. HEALTH PROVIDERS** (includes medical providers, dentists and dental specialists)					**3. FRIENDS/OTHER PEOPLE** (includes friends, neighbors, co-workers etc. anyone that is not family or health providers)				
	**Never 0**	**Rarely 1**	**Sometimes 2**	**Often 3**	**Always 4**	**Never 0**	**Rarely 1**	**Sometimes 2**	**Often 3**	**Always 4**	**Never 0**	**Rarely 1**	**Sometimes 2**	**Often 3**	**Always 4**
EXAMPLE: They make sure I get my flu shot.				X			X				X				

BRUSHING YOUR TEETH

In this section, think about help or support you get with brushing your teeth (cleaning them, using either a manual or electric toothbrush, or other similar tool).

Remember to rate each statement 3 times (from left to right →). Make sure each group has a rating.
If the statement does not apply to any of the groups for you, then please check "Never."

For each statement, how often do you get support from each group of people for BRUSHING YOUR TEETH?	1. FAMILY (includes your significant other, and any immediate and extended family members you may or may not live with)					2. HEALTH PROVIDERS (includes medical providers, dentists and dental specialists)					3. FRIENDS/OTHER PEOPLE (includes friends, neighbors, co-workers; anyone that is not family or health providers)				
	Never 0	Rarely 1	Sometimes 2	Often 3	Always 4	Never 0	Rarely 1	Sometimes 2	Often 3	Always 4	Never 0	Rarely 1	Sometimes 2	Often 3	Always 4
1. They show me how to brush my teeth. (brush1)															
2. They make sure I have a toothbrush. (brush2)															
3. They explain how to brush my teeth correctly. (brush5)															
4. They remind me to get more dental supplies (for example, toothbrush, toothpaste, floss, etc.). (brush10)															
5. They tell me that brushing my teeth is important to my health. (brush12)															

For each statement, how often do you get support from each group of people for **BRUSHING YOUR TEETH?**	**1. FAMILY** (includes your significant other, and any immediate and extended family members you may or may not live with)					**2. HEALTH PROVIDERS** (includes medical providers, dentists and dental specialists)					**3. FRIENDS/OTHER PEOPLE** (includes friends, neighbors, co-workers; anyone that is not family or health providers)				
	Never 0	Rarely 1	Sometimes 2	Often 3	Always 4	Never 0	Rarely 1	Sometimes 2	Often 3	Always 4	Never 0	Rarely 1	Sometimes 2	Often 3	Always 4
6. They tell me to brush my teeth after meals. (brush14)															
7. They tell me to brush my teeth regularly (at least twice a day). (brush15)															
8. They tell me what will happen if I do not brush my teeth regularly. (brush16)															
9. They explain to me why my gums might bleed during or after brushing my teeth. (brush17)															
10. They help me feel confident in my ability to brush my teeth. (brush19)															
11. They encourage me to brush my teeth. (brush22)															
12. They tell me I brush my teeth well. (brush24)															

FLOSSING

In this section, think about help or support you get with flossing, (cleaning in between your teeth, using other tools like toothpicks, soft picks, waterpik, etc.).

Remember to rate each question 3 times (from left to right →). Make sure each group has a rating.
If the statement does not apply to any of the groups for you, then please check "Never."

**For each statement, how often do you get support from each group of people for FLOSSING?**	**1. FAMILY (includes your significant other, and any immediate and extended family members you may or may not live with)**					**2. HEALTH PROVIDERS (includes medical providers, dentists and dental specialists)**					**3. FRIENDS/OTHER PEOPLE (includes friends, neighbors, co-workers; anyone that is not family or health providers)**				
	**Never 0**	**Rarely 1**	**Sometimes 2**	**Often 3**	**Always 4**	**Never 0**	**Rarely 1**	**Sometimes 2**	**Often 3**	**Always 4**	**Never 0**	**Rarely 1**	**Sometimes 2**	**Often 3**	**Always 4**
13. They show me how to floss my teeth. (floss28)															
14. They make sure I have floss. (floss29)															
15. They explain how to floss my teeth correctly. (floss31)															
16. They tell me that flossing my teeth is important to my health. (floss33)															
17. They tell me to floss my teeth after meals. (floss34)															

For each statement, how often do you get support from each group of people for FLOSSING?	1. FAMILY (includes your significant other, and any immediate and extended family members you may or may not live with)					2. HEALTH PROVIDERS (includes medical providers, dentists and dental specialists)					3. FRIENDS/OTHER PEOPLE (includes friends, neighbors, co-workers; anyone that is not family or health providers)				
	Never 0	Rarely 1	Sometimes 2	Often 3	Always 4	Never 0	Rarely 1	Sometimes 2	Often 3	Always 4	Never 0	Rarely 1	Sometimes 2	Often 3	Always 4
18. They tell me to floss my teeth regularly (at least once a day). (floss35)															
19. They tell what will happen if I do not floss my teeth regularly. (floss36)															
20. They explain to me why my gums might bleed during or after I use floss. (floss37)															
21. They help me feel confident in my ability to floss my teeth. (floss39)															
22. They encourage me to floss my teeth. (floss42)															
23. They tell me I floss my teeth well. (floss44)															

GETTING DENTAL CARE

In this section, think about help or support you get for getting dental care. This can include finding dentists, making and getting to your dental appointments, and deciding to get dental care or treatments. We are interested in all dental care you get, so think about all types of dental services, including dental exams, check-ups, cleanings, and any other type of dental treatments (fillings, extractions, braces, crowns, bridges, implants, etc.). Please answer about support you get for dental care now, as an adult.

Remember to rate each question 3 times (from left to right →). Make sure each group has a rating.
If the statement does not apply to any of the groups for you, then please check "Never."

For each statement, how often do you get support from each group of people for GETTING DENTAL CARE?	1. FAMILY (includes your significant other, and any immediate and extended family members you may or may not live with)					2. HEALTH PROVIDERS (includes medical providers, dentists and dental specialists)					3. FRIENDS/OTHER PEOPLE (includes friends, neighbors, co-workers; anyone that is not family or health providers)				
	Never 0	Rarely 1	Sometimes 2	Often 3	Always 4	Never 0	Rarely 1	Sometimes 2	Often 3	Always 4	Never 0	Rarely 1	Sometimes 2	Often 3	Always 4
24. They help me manage challenges or obstacles to getting dental care. (dentalcare47)															
25. They help me get dental care.(dentalcare53)															
26. They help me find a dentist that meets my needs.(dentalcare57)															

For each statement, how often do you get support from each group of people for <u>GETTING DENTAL CARE?</u>	1. FAMILY (includes your significant other, and any immediate and extended family members you may or may not live with)					2. HEALTH PROVIDERS (includes medical providers, dentists and dental specialists)					3. FRIENDS/OTHER PEOPLE (includes friends, neighbors, co-workers; anyone that is not family or health providers)				
	Never 0	Rarely 1	Sometimes 2	Often 3	Always 4	Never 0	Rarely 1	Sometimes 2	Often 3	Always 4	Never 0	Rarely 1	Sometimes 2	Often 3	Always 4
27. They remind me about my dental appointment. (dentalcare64)															
28. They tell me what will happen during my dental treatment. (dentalcare67)															
29. They answer questions about my dental care. (dentalcare68)															
30. They tell me that going to the dentist is important to my health. (dentalcare69)															
31. They explain why the dental treatment is needed. (dentalcare71)															
32. They listen to my dental care concerns. (dentalcare79)															
33. They talk me through my dental treatment options. (dentalcare83)															

For each statement, how often do you get support from each group of people for **GETTING DENTAL CARE?**	**1. FAMILY** (includes your significant other, and any immediate and extended family members you may or may not live with)					**2. HEALTH PROVIDERS** (includes medical providers, dentists and dental specialists)					**3. FRIENDS/OTHER PEOPLE** (includes friends, neighbors, co-workers; anyone that is not family or health providers)				
	Never 0	Rarely 1	Sometimes 2	Often 3	Always 4	Never 0	Rarely 1	Sometimes 2	Often 3	Always 4	Never 0	Rarely 1	Sometimes 2	Often 3	Always 4
34. They tell me to go to the dentist regularly. (dentalcare86)															
35. They tell me that everyone goes to the dentist. (dentalcare88)															
36. They help me change how I take care of my teeth. (dentalcare92)															
37. They help me make changes to what I eat and drink for my dental health. (dentalcare93)															
38. They tell me to go to the dentist for my dental problems or discomfort. (dentalcare97)															
39. They follow up or check-in after dental treatment.(dentalcare101)															

OPTIONAL SUBSCALES – IF APPLY – up to 10 items (dropped possible medication/major tx items)

Please answer this last set of questions about getting dental care, if they apply to you.

Language Help

Do you need help from an interpreter/translator when getting dental care? Select one: Yes or No.
If yes, then rate:

	2. FAMILY (includes your significant other, and any immediate and extended family members you may or may not live with)					3. HEALTH PROVIDERS (includes medical providers, dentists and dental specialists)					4. FRIENDS/OTHER PEOPLE (includes friends, neighbors, co-workers; anyone that is not family or health providers)				
	Never 0	Rarely 1	Sometimes 2	Often 3	Always 4	Never 0	Rarely 1	Sometimes 2	Often 3	Always 4	Never 0	Rarely 1	Sometimes 2	Often 3	Always 4
They interpret (translate) or get an interpreter (translator) for me. (dentalcare50)															

Transportation Help

Do you need help getting to or from dental appointments? Select one: Yes or No.

If yes, then rate:

	1. FAMILY (includes your significant other, and any immediate and extended family members you may or may not live with)					2. HEALTH PROVIDERS (includes medical providers, dentists and dental specialists)					3. FRIENDS/OTHER PEOPLE (includes friends, neighbors, co-workers; anyone that is not family or health providers)				
	Never 0	Rarely 1	Sometimes 2	Often 3	Always 4	Never 0	Rarely 1	Sometimes 2	Often 3	Always 4	Never 0	Rarely 1	Sometimes 2	Often 3	Always 4
They help me get to/from the dentist (for example, give me a ride, or arrange a ride). (dentalcare49)															

Help Paying

Do you need help paying for anything related to dental care? Select one: Yes or No.

If yes, then rate:

	**1. FAMILY** (includes your significant other, and any immediate and extended family members you may or may not live with)					**2. HEALTH PROVIDERS** (includes medical providers, dentists and dental specialists)					**3. FRIENDS/OTHER PEOPLE** (includes friends, neighbors, co-workers; anyone that is not family or health providers)				
	Never 0	Rarely 1	Sometimes 2	Often 3	Always 4	Never 0	Rarely 1	Sometimes 2	Often 3	Always 4	Never 0	Rarely 1	Sometimes 2	Often 3	Always 4
They give me information on dental insurance. (dentalcare76)															
They explain what my dental insurance covers. (dentalcare72)															
They help me pay for dental care (for example, pay for me, offer me a payment plan, lend me the money). (dentalcare48)															

Dental Fears/Worries

Do you have any dental fears or worries? Select one: Yes or No.

If yes, then rate:

	1. FAMILY (includes your significant other, and any immediate and extended family members you may or may not live with)					2. HEALTH PROVIDERS (includes medical providers, dentists and dental specialists)					3. FRIENDS/OTHER PEOPLE (includes friends, neighbors, co-workers; anyone that is not family or health providers)				
	Never 0	Rarely 1	Sometimes 2	Often 3	Always 4	Never 0	Rarely 1	Sometimes 2	Often 3	Always 4	Never 0	Rarely 1	Sometimes 2	Often 3	Always 4
They listen to my fears about going to the dentist. (dentalcare80)															
They listen to any worries about going to the dentist. (dentalcare82)															
They ease my worries about dental treatments. (dentalcare103)															
They ease my fears about dental treatments. (dentalcare104)															

[Attention Check Question]

Please select option B:
a) smile b) teeth c) toothbrush

Encuesta de Apoyo de Salud Dental – OHBSS, v3.17.2022

INSTRUCCIONES:

Esta encuesta pregunta acerca de la ayuda o el apoyo social que recibe de otras personas en este momento para diferentes comportamientos de salud dental. Por favor, responda cada pregunta <u>3 veces</u> - una vez para cada grupo de personas.

1) **Familia:** incluye a su pareja, y miembros de su familia que habiten o no habiten con usted
2) **Proveedores de salud:** incluye proveedores médicos, dentistas y otros especialistas dentales
3) **Amigos y otra gente:** incluye amigos, vecinos, compañeros de trabajo etc.

Por favor, piense y responda las preguntas en base a sus experiencias dentales <u>actuales</u> y comportamientos que haga <u>ahora</u>, como adulto. No hay respuestas correctas o incorrectas. Estamos interesados en quién le da apoyo ahorita. Seleccione las mejores respuestas para cada pregunta y no deje nada en blanco.

Por favor, mire el ejemplo a continuación. Recuerde responder cada pregunta <u>3 veces</u> (de izquierda a derecha →) y asegúrese de que cada grupo tenga una respuesta.
Si la pregunta no aplica a ninguno de los grupos para usted, por favor responda "NUNCA."

**Para cada pregunta, ¿con qué frecuencia recibe apoyo de cada grupo de personas?**	**1. FAMILIA** **(incluye a su pareja, y miembros de su familia que habiten o no habiten con usted)**					**2. PROVEEDORES DE SALUD** **(incluye proveedores médicos, dentistas y otros especialistas dentales)**					**3. AMIGOS/OTRA GENTE** **(incluye amigos, vecinos, compañeros de trabajo etc. Cualquier persona que no sea familia o proveedores de salud)**				
	**Nunca$_0$**	**Raramente $_1$**	**A Veces $_2$**	**A Menudo $_3$**	**Siempre$_4$**	**Nunca$_0$**	**Raramente $_1$**	**A Veces $_2$**	**A Menudo $_3$**	**Siempre$_4$**	**Nunca$_0$**	**Raramente $_1$**	**A Veces $_2$**	**A Menudo $_3$**	**Siempre$_4$**
EJEMPLO: Se aseguran de que reciba mi inyección para la gripe.				X			X				X				

CEPILLARSE LOS DIENTES

En esta sección, piense en la ayuda o el apoyo que recibe al cepillarse los dientes (al limpiarlos, usando un cepillo de dientes manual o eléctrico u otra herramienta similar).

Recuerde responder cada pregunta 3 veces (de izquierda a derecha →), asegúrese de que cada grupo tenga una respuesta.
Si la pregunta no aplica a ninguno de los grupos para usted, por favor responda "NUNCA."

**Para cada pregunta, ¿con qué frecuencia recibe apoyo de cada grupo de personas al CEPILLARSE LOS DIENTES?**	**1. FAMILIA (incluye a su pareja, y miembros de su familia que habiten o no habiten con usted)**					**2. PROVEEDORES DE SALUD (incluye proveedores médicos, dentistas y otros especialistas dentales)**					**3. AMIGOS/OTRA GENTE (incluye amigos, vecinos, compañeros de trabajo etc. Cualquier persona que no sea familia o proveedores de salud)**				
	**Nunca 0**	**Raramente 1**	**A Veces 2**	**A Menudo 3**	**Siempre 4**	**Nunca 0**	**Raramente 1**	**A Veces 2**	**A Menudo 3**	**Siempre 4**	**Nunca 0**	**Raramente 1**	**A Veces 2**	**A Menudo 3**	**Siempre 4**
1. Me enseñan a cepillarme los dientes.															
2. Se aseguran que tengo un cepillo de dientes.															
3. Me explican cómo cepillarme los dientes correctamente.															
4. Me recuerdan de conseguir más productos dentales (por ejemplo, cepillo, pasta, hilo dental, etc.)															

Para cada pregunta, ¿con qué frecuencia recibe apoyo de cada grupo de personas al **CEPILLARSE LOS DIENTES?**	**1. FAMILIA** (incluye a su pareja, y miembros de su familia que habiten o no habiten con usted)					**2. PROVEEDORES DE SALUD** (incluye proveedores médicos, dentistas y otros especialistas dentales)					**3. AMIGOS/OTRA GENTE** (incluye amigos, vecinos, compañeros de trabajo etc. Cualquier persona que no sea familia o proveedores de salud)				
	Nunca 0	Raramente 1	A Veces 2	A Menudo 3	Siempre 4	Nunca 0	Raramente 1	A Veces 2	A Menudo 3	Siempre 4	Nunca 0	Raramente 1	A Veces 2	A Menudo 3	Siempre 4
5. Me dicen que cepillarme los dientes es importante para mi salud.															
6. Me dicen que me cepille los dientes después de cada comida.															
7. Dicen que me cepille los dientes de manera regular (al menos dos veces al día).															
8. Me dicen que pasará si no me cepillo los dientes de manera regular.															
9. Me explican porque me pueden sangrar las encías durante o después de cepillarme los dientes.															
10. Me ayudan a sentirme seguro en mi forma de cepillarme los dientes.															

Para cada pregunta, ¿con qué frecuencia recibe apoyo de cada grupo de personas al **CEPILLARSE LOS DIENTES?**	**1. FAMILIA** (incluye a su pareja, y miembros de su familia que habiten o no habiten con usted)					**2. PROVEEDORES DE SALUD** (incluye proveedores médicos, dentistas y otros especialistas dentales)					**3. AMIGOS/OTRA GENTE** (incluye amigos, vecinos, compañeros de trabajo etc. Cualquier persona que no sea familia o proveedores de salud)				
	Nunca $_0$	Raramente $_1$	A Veces $_2$	A Menudo $_3$	Siempre $_4$	Nunca $_0$	Raramente $_1$	A Veces $_2$	A Menudo $_3$	Siempre $_4$	Nunca $_0$	Raramente $_1$	A Veces $_2$	A Menudo $_3$	Siempre $_4$
11. Me motivan a cepillarme los dientes.															
12. Me dicen que me cepillo los dientes bien.															

USAR HILO DENTAL (LIMPIEZA ENTRE LOS DIENTES)

En esta sección, piense en la ayuda o el apoyo que recibe al usar hilo dental, (limpiar entre los dientes, usar otras herramientas como palillos, palillos blandos, waterpik, etc.).

Recuerde responder cada pregunta 3 veces (de izquierda a derecha →), asegúrese de que cada grupo tenga una respuesta.
Si la pregunta no aplica a ninguno de los grupos para usted, por favor responda "NUNCA".

**Para cada pregunta, ¿con qué frecuencia recibe apoyo de cada grupo de personas al USAR HILO DENTAL?**	**1. FAMILIA (incluye a su pareja, y miembros de su familia que habiten o no habiten con usted)**					**2. PROVEEDORES DE SALUD (incluye proveedores médicos, dentistas y otros especialistas dentales)**					**3. AMIGOS/OTRA GENTE (incluye amigos, vecinos, compañeros de trabajo etc. Cualquier persona que no sea familia o proveedores de salud)**				
	Nunca$_0$	Raramente $_1$	A Veces $_2$	A Menudo $_3$	Siempre$_4$	Nunca$_0$	Raramente $_1$	A Veces $_2$	A Menudo $_3$	Siempre$_4$	Nunca$_0$	Raramente $_1$	A Veces $_2$	A Menudo $_3$	Siempre$_4$
13. Me enseñan a usar hilo dental.															
14. Se aseguran que tengo hilo dental.															
15. Me explican cómo usar hilo dental correctamente.															
16. Me dicen que usar hilo dental es importante para mi salud.															
17. Me dicen que use hilo dental después de cada comida.															

**Para cada pregunta, ¿con qué frecuencia recibe apoyo de cada grupo de personas al USAR HILO DENTAL?**	**1. FAMILIA (incluye a su pareja, y miembros de su familia que habiten o no habiten con usted)**					**2. PROVEEDORES DE SALUD (incluye proveedores médicos, dentistas y otros especialistas dentales)**					**3. AMIGOS/OTRA GENTE (incluye amigos, vecinos, compañeros de trabajo etc. Cualquier persona que no sea familia o proveedores de salud)**				
	Nunca$_0$	Raramente $_1$	A Veces $_2$	A Menudo $_3$	Siempre$_4$	Nunca$_0$	Raramente $_1$	A Veces $_2$	A Menudo $_3$	Siempre$_4$	Nunca$_0$	Raramente $_1$	A Veces $_2$	A Menudo $_3$	Siempre$_4$
18. Me dicen que use hilo dental regularmente (al menos una vez al día).															
19. Me dicen qué pasa si no uso hilo dental regularmente.															
20. Me explican porque me pueden sangrar las encías durante o después de usar hilo dental.															
21. Me ayudan a sentirme seguro en mi forma de usar hilo dental.															
22. Me motivan a usar hilo dental.															
23. Me dicen que uso bien el hilo dental.															

OBTENER CUIDADO DENTAL

En esta sección, piense en la ayuda o el apoyo que recibe al recibir atención dental. Esto puede incluir ayuda con buscar dentistas, hacer citas dentales, llegar a citas dentales, o a decidir si necesita atención o tratamientos dentales. Estamos interesados en todo el cuidado dental que recibe, así que piense en todo tipo de servicios dentales, incluyendo exámenes dentales, chequeos, limpiezas, y cualquier otro tipo de tratamientos dentales (rellenos, extracciones, frenos/brackets dentales, coronas, puentes, implantes, etc.). Por favor, responda sobre el apoyo que recibe para la atención dental ahora, como adulto.

Recuerde responder cada pregunta 3 veces (de izquierda a derecha →). Asegúrese de que cada grupo tenga una respuesta.
Si la pregunta no aplica a ninguno de los grupos para usted, por favor responda "Nunca".

**Para cada pregunta, ¿con qué frecuencia recibe apoyo de cada grupo de personas al RECIBIR ATENCIÓN DENTAL?**	**1. FAMILIA (incluye a su pareja, y miembros de su familia que habiten o no habiten con usted)**					**2. PROVEEDORES DE SALUD (incluye proveedores médicos, dentistas y otros especialistas dentales)**					**3. AMIGOS/OTRA GENTE (incluye amigos, vecinos, compañeros de trabajo etc. Cualquier persona que no sea familia o proveedores de salud)**				
	Nunca 0	Raramente 1	A Veces 2	A Menudo 3	Siempre 4	Nunca 0	Raramente 1	A Veces 2	A Menudo 3	Siempre 4	Nunca 0	Raramente 1	A Veces 2	A Menudo 3	Siempre 4
24. Me ayudan a solucionar problemas para poder tener cuidado dental.															
25. Me ayudan a conseguir cuidado dental.															
26. Me ayudan a buscar un dentista que cumple mis necesidades.															

Para cada pregunta, ¿con qué frecuencia recibe apoyo de cada grupo de personas al <u>RECIBIR ATENCIÓN DENTAL?</u>	**1. FAMILIA** (incluye a su pareja, y miembros de su familia que habiten o no habiten con usted)					**2. PROVEEDORES DE SALUD** (incluye proveedores médicos, dentistas y otros especialistas dentales)					**3. AMIGOS/OTRA GENTE** (incluye amigos, vecinos, compañeros de trabajo etc. Cualquier persona que no sea familia o proveedores de salud)				
	Nunca $_0$	Raramente $_1$	A Veces $_2$	A Menudo $_3$	Siempre $_4$	Nunca $_0$	Raramente $_1$	A Veces $_2$	A Menudo $_3$	Siempre $_4$	Nunca $_0$	Raramente $_1$	A Veces $_2$	A Menudo $_3$	Siempre $_4$
27. Me recuerdan de mi cita dental.															
28. Me dicen lo que pasará durante mi tratamiento dental.															
29. Contestan preguntas sobre mi cuidado dental.															
30. Me dicen que ir al dentista es importante para mi salud.															
31. Me explican por qué el tratamiento dental es necesario.															
32. Escuchan mis preocupaciones sobre mi cuidado dental.															
33. Me hablan de las opciones que tengo para mí tratamiento dental.															

Para cada pregunta, ¿con qué frecuencia recibe apoyo de cada grupo de personas al **RECIBIR ATENCIÓN DENTAL?**	**1. FAMILIA** (incluye a su pareja, y miembros de su familia que habiten o no habiten con usted)					**2. PROVEEDORES DE SALUD** (incluye proveedores médicos, dentistas y otros especialistas dentales)					**3. AMIGOS/OTRA GENTE** (incluye amigos, vecinos, compañeros de trabajo etc. Cualquier persona que no sea familia o proveedores de salud)				
	Nunca $_0$	Raramente $_1$	A Veces $_2$	A Menudo $_3$	Siempre $_4$	Nunca $_0$	Raramente $_1$	A Veces $_2$	A Menudo $_3$	Siempre $_4$	Nunca $_0$	Raramente $_1$	A Veces $_2$	A Menudo $_3$	Siempre $_4$
34. Me dicen que vaya al dentista de manera regular.															
35. Me dicen que todos van al dentista.															

36. Me ayudan a cambiar como me cuido los dientes.															
37. Me ayudan a lograr cambios en lo que como y bebo para mi salud dental.															
38. Me dicen que vaya al dentista con respecto a mis problemas o molestias dentales.															
39. Me dan seguimiento después de un tratamiento dental.															

OPTIONAL SUBSCALES – IF APPLY – up to 10 items (dropped possible medication/major tx items)

Por favor responda a esta última serie de preguntas.

Ayuda con Lenguaje

¿Necesita ayuda de un intérprete/traductor cuando recibe atención dental? Seleccione uno: Sí o No.
En caso afirmativo, califique:

	1. FAMILIA (incluye a su pareja, y miembros de su familia que habiten o no habiten con usted)					2. PROVEEDORES DE SALUD (incluye proveedores médicos, dentistas y otros especialistas dentales)					3. AMIGOS/OTRA GENTE (incluye amigos, vecinos, compañeros de trabajo etc. Cualquier persona que no sea familia o proveedores de salud)				
	Nunca 0	Raramente 1	A Veces 2	Nunca 0	Raramente 1	A Veces 2	Nunca 0	Raramente 1	A Veces 2	Nunca 0	Raramente 1	A Veces 2	Nunca 0	Raramente 1	A Veces 2
Interpretan (traducen) o me consiguen un intérprete (traductor).															

Ayuda con Transporte

¿Necesita ayuda para ir o volver de las citas con el dentista? Seleccione uno: Sí o No.
En caso afirmativo, califique:

	**2. FAMILIA** (incluye a su pareja, y miembros de su familia que habiten o no habiten con usted)					**2. PROVEEDORES DE SALUD** (incluye proveedores médicos, dentistas y otros especialistas dentales)					**3. AMIGOS/OTRA GENTE** (incluye amigos, vecinos, compañeros de trabajo etc. Cualquier persona que no sea familia o proveedores de salud)				
	Nunca $_0$	Raramente $_1$	A Veces $_2$	Nunca $_0$	Raramente $_1$	A Veces $_2$	Nunca $_0$	Raramente $_1$	A Veces $_2$	Nunca $_0$	Raramente $_1$	A Veces $_2$	Nunca $_0$	Raramente $_1$	A Veces $_2$
Me ayudan a ir/venir del dentista (por ejemplo, me llevan, o me consiguen transporte).															

Ayuda para pagar

¿Necesita ayuda para pagar algo relacionado con el cuidado dental?
Seleccione uno: Sí o No.
En caso afirmativo, califique:

	**3. FAMILIA** (incluye a su pareja, y miembros de su familia que habiten o no habiten con usted)					**2. PROVEEDORES DE SALUD** (incluye proveedores médicos, dentistas y otros especialistas dentales)					**3. AMIGOS/OTRA GENTE** (incluye amigos, vecinos, compañeros de trabajo etc. Cualquier persona que no sea familia o proveedores de salud)				
	Nunca$_0$	Raramente $_1$	A Veces $_2$	Nunca$_0$	Raramente $_1$	A Veces $_2$	Nunca$_0$	Raramente $_1$	A Veces $_2$	Nunca$_0$	Raramente $_1$	A Veces $_2$	Nunca$_0$	Raramente $_1$	A Veces $_2$
Me dan información sobre el seguro dental (aseguranza).															
Me ayudan con el seguro dental (aseguranza).															
Me ayudan a pagar por el dentista (por ejemplo, pagan por mí, me dan opciones de pago, me hacen un préstamo).															

Miedo dental/preocupación

¿Tiene algún miedo o preocupación dental?
Seleccione uno: Sí o No.
En caso afirmativo, califique:

	**4. FAMILIA** (incluye a su pareja, y miembros de su familia que habiten o no habiten con usted)					**2. PROVEEDORES DE SALUD** (incluye proveedores médicos, dentistas y otros especialistas dentales)					**3. AMIGOS/OTRA GENTE** (incluye amigos, vecinos, compañeros de trabajo etc. Cualquier persona que no sea familia o proveedores de salud)				
	Nunca $_0$	Raramente $_1$	A Veces $_2$	Nunca $_0$	Raramente $_1$	A Veces $_2$	Nunca $_0$	Raramente $_1$	A Veces $_2$	Nunca $_0$	Raramente $_1$	A Veces $_2$	Nunca $_0$	Raramente $_1$	A Veces $_2$
Escuchan mis preocupaciones sobre ir al dentista.															
Escuchan cualquier preocupación relacionada con ir al dentista.															
Calman mis preocupaciones sobre los tratamientos dentales.															
Calman mis miedos sobre los tratamientos dentales.															

[Attention check Question - Por favor seleccione la opción B:

a) sonrisa b) dientes c) cepillo dental]
