# Supplementary material for: Development of new bilingual oral health behavior social support (OHBSS) scales in English and Spanish
Source: PLoS One. 2025 Mar 11;20(3):e0317133. doi: 10.1371/journal.pone.0317133 (PMC11896079; doi:10.1371/journal.pone.0317133)
Supplement: S6 Table — (PDF) [file pone.0317133.s006.pdf]

Supplement 6 Table. Items in final OHBSS scales with type of social support, reading ease scores and variable names

					SOURCES OF SOCIAL SUPPORT		
**Type of Social Support**	**B = BRUSHING SOCIAL SUPPORT SCALE - ENGLISH ITEMS**	**Flesch-Kincaid Reading Ease score (Grade Level)**	**B = BRUSHING SOCIAL SUPPORT SCALE - SPANISH ITEMS**	**Fernandez-Huerta Reading Ease score (Grade Level)**	**Family (F)**	**Health Providers (P)**	**Others/Friends (O)**
Instrumental	They show me how to brush my teeth.	100 (0.0)	Me enseñan a cepillarme los dientes.	80.72 (5.0)	BF1	BP1	BO1
Instrumental	They make sure I have a toothbrush.	100 (0.6)	Se aseguran que tengo un cepillo de dientes.	89.96 (5.0)	BF2	BP2	BO2
Instrumental	They explain how to brush my teeth correctly.	82.3 (3.7)	Me explican cómo cepillarme los dientes correctamente.	49.07 (Difícil (cursos selectivos))	BF3	BP3	BO3
Informational*	They remind me to get more dental supplies (for example, toothbrush, toothpaste, floss, etc.)	65.7 (7.5)	Me recuerdan de conseguir más productos dentales (por ejemplo, cepillo, pasta, hilo dental, etc.	73.68 (6.0)	BF4	BP4	BO4
Informational	They tell me that brushing my teeth is important to my health.	88.9 (3.8)	Me dicen que cepillarme los dientes es importante para mi salud.	86.63 (5.0)	BF5	BP5	BO5
Informational	They tell me to brush my teeth after meals.	100 (0.0)	Me dicen que me cepille los dientes después de cada comida.	97.43 (4.0)	BF6	BP6	BO6
Informational	They tell me to brush my teeth regularly (at least twice a day).	89.5 (4.0)	Dicen que me cepille los dientes regularmente (al menos dos veces al día).	94.68 (4.0)	BF7	BP7	BO7
Informational	They tell me what will happen if I do not brush my teeth regularly.	83.8 (5.0)	Me dicen que pasará si no me cepillo los dientes de manera regular.	94.01 (4.0)	BF8	BP8	BO8
Informational	They explain to me why my gums might bleed during or after brushing my teeth.	84.4 (5.2)	Me explican porque me pueden sangrar las encías durante o después de cepillarme los dientes.	83.39 (5.0)	BF9	BP9	BO9
Informational	They help me feel confident in my ability to brush my teeth.	74.8 (5.8)	Me ayudan a sentirme seguro en mi forma de cepillarme los dientes.	85.52 (5.0)	BF10	BP10	BO10
Emotional	They encourage me to brush my teeth.	90.9 (2.3)	Me motivan a cepillarme los dientes.	80.72 (5.0)	BF11	BP11	BO11
Appraisal	They tell me I brush my teeth well.	100 (0.0)	Me dicen que me cepillo los dientes bien.	112.76 (4.0)	BF12	BP12	BO12
	**F = FLOSSING SOCIAL SUPPORT SCALE - ENGLISH ITEMS**		**F = FLOSSING SOCIAL SUPPORT SCALE - SPANISH ITEMS**				
Instrumental	They show me how to floss my teeth.	100 (0.0)	Me enseñan a usar hilo dental.	93.98 (4.0)	FF1	FP1	FO1
Instrumental	They make sure I have floss.	100 (0.0)	Se aseguran que tengo hilo dental.	80.72 (5.0)	FF2	FP2	FO2
Instrumental	They explain how to floss my teeth correctly.	82.3 (3.7)	Me explican cómo usar hilo dental correctamente.	57.47 (Pre-universitario (algo difícil))	FF3	FP3	FO3
Informational*	They remind me to get more dental supplies (for example, toothbrush, toothpaste, floss, etc.)	65.7 (7.5)	Me recuerdan de conseguir más productos dentales (por ejemplo, cepillo, pasta, hilo dental, etc.	73.68 (6.0)	FF4	FP4	FO4
Informational	They tell me that flossing my teeth is important to my health.	88.9 (3.8)	Me dicen que usar hilo dental es importante para mi salud.	86.42 (5.0)	FF5	FP5	FO5
Informational	They tell me to floss my teeth after meals.	100 (0.0)	Me dicen que use hilo dental después de cada comida.	93.74 (4.0)	FF6	FP6	FO6
Informational	They tell me to floss my teeth regularly (at least once a day).	89.5 (4.0)	Me dicen que use hilo dental regularmente (al menos una vez al día).	91.42 (4.0)	FF7	FP7	FO7
Informational	They tell what will happen if I do not floss my teeth regularly.	83 (4.9)	Me dicen qué pasa si no uso hilo dental regularmente.	87.74 (5.0)	FF8	FP8	FO8
Informational	They explain to me why my gums might bleed during or after I use floss.	90 (4.4)	Me explican porque me pueden sangrar las encías durante o después de usar hilo dental.	86.99 (5.0)	FF9	FP9	FO9
Informational	They help me feel confident in my ability to floss my teeth.	74.8 (5.8)	Me ayudan a sentirme seguro en mi forma de usar hilo dental.	90.92 (4.0)	FF10	FP10	FO10
Emotional	They encourage me to floss my teeth.	90.9 (2.3)	Me motivan a usar hilo dental.	93.98 (4.0)	FF11	FP11	FO11
Appraisal	They tell me I floss my teeth well.	100 (0.0)	Me dicen que uso bien el hilo dental.	112.76 (4.0)	FF12	FP12	FO12
*Item is asked once, but scored with both brushing (B) and flossing (F) scales, so each is 12 items							
	**D = DENTAL CARE SOCIAL SUPPORT SCALE - ENGLISH ITEMS (REQUIRED SET)**		**D = DENTAL CARE SOCIAL SUPPORT SCALE - SPANISH ITEMS (REQUIRED SET)**				
Instrumental	They help me get dental care.	100 (0.5)	Me ayudan a conseguir cuidado dental.	73.58 (6.0)	DF1	DP1	DO1
Instrumental	They help me find a dentist that meets my needs.	100 (1.2)	Me ayudan a buscar un dentista que cumple mis necesidades.	81.74 (5.0)	DF2	DP2	DO2
Informational	They remind me about my dental appointment.	54.7 (7.3)	Me recuerdan de mi cita dental.	103.58 (4.0)	DF3	DP3	DO3
Informational	They tell me what will happen during my dental treatment.	78.2 (4.8)	Me dicen lo que pasará durante mi tratamiento dental.	82.25 (5.0)	DF4	DP4	DO4
Informational	They answer questions about my dental care.	66.7 (5.6)	Contestan preguntas sobre mi cuidado dental.	63.98 (7.0/8.0)	DF5	DP5	DO5
Informational	They tell me that going to the dentist is important to my health.	89.5 (4.0)	Me dicen que ir al dentista es importante para mi salud.	97.43 (4.0)	DF6	DP6	DO6
Informational	They explain why the dental treatment is needed.	71.8 (5.2)	Me explican por qué el tratamiento dental es necesario.	82.25 (5.0)	DF7	DP7	DO7
Emotional	They listen to my dental care concerns.	78.8 (3.9)	Escuchan mis preocupaciones sobre mi cuidado dental.	49.07 (Cursos Selectivos (difícil))	DF8	DP8	DO8
Appraisal	They tell me to go to the dentist regularly.	79.3 (4.4)	Me dicen que vaya al dentista de manera regular.	88.85 (5.0)	DF9	DP9	DO9
Informational	They tell me that everyone goes to the dentist.	84.9 (3.6)	Me dicen que todos van al dentista.	109.07 (4.0)	DF10	DP10	DO10
Instrumental	They help me change how I take care of my teeth.	100 (0.5)	Me ayudan a cambiar como me cuido los dientes.	102.05 (4.0)	DF11	DP11	DO11
Instrumental	They help me make changes to what I eat and drink for my dental health.	95.7 (3.6)	Me ayudan a lograr cambios en lo que como y bebo para mi salud dental.	103.19 (4.0)	DF12	DP12	DO12
Informational	They tell me to go to the dentist for my dental problems or discomfort.	77.8 (5.8)	Me dicen que vaya al dentista con respecto a mis problemas o molestias dentales.	88.1 (5.0)	DF13	DP13	DO13
Instrumental	They follow up or check-in after dental treatment.	61.2 (6.7)	Me dan seguimiento después de un tratamiento dental.	82.76 (5.0)	DF14	DP14	DO14
	**OPTIONAL SET OF DENTAL CARE SOCIAL SUPPORT SCALE - ENGLISH ITEMS**		**OPTIONAL SET OF DENTAL CARE SOCIAL SUPPORT SCALE - SPANISH ITEMS**				
	**Please answer this last set of questions about getting dental care, if they apply to you.**		**Por favor responda a esta última serie de preguntas.**				
	**TRANSLATE = Translation help needed for dental care**		**Ayuda con Lenguaje**				
	Do you need help from an interpreter/translator when getting dental care?	59.3 (8.0)	¿Necesita ayuda de un intérprete/traductor cuando recibe atención dental?	45.74 (difícil)			
Instrumental	They interpret (translate) or get an interpreter (translator) for me.	44.4 (9.6)	Interpretan (traducen) o me consiguen un intérprete (traductor).	55.21 (algo difícil)	TranslateF	TranslateP	TranslateO
	**TRANSPORT = Transportation help needed for dental care**		**Ayuda con Transporte**				
	Do you need help getting to or from dental appointments?	78.2 (4.8)	¿Necesita ayuda para ir o volver de las citas con el dentista?	90.92 (4.2)			
Instrumental	They help me get to/from the dentist (for example, give me a ride, or arrange a ride).	85.1 (5.8)	Me ayudan a ir/venir del dentista (por ejemplo, me llevan, o me consiguen transporte).	93.74 (4.0)	TransportF	TransportP	TransportO
	**PAY = Help needed to pay for dental care**		**Ayuda para pagar**				
	Do you need help paying for anything related to dental care?	68.8 (6.4)	¿Necesita ayuda para pagar algo relacionado con el cuidado dental?	51.74 (algo difícil)			
informational	They give me information on dental insurance.	42.6 (9.0)	Me dan información sobre el seguro dental (aseguranza).	61.32 (7.0/8.0)	Pay1F	Pay1P	Pay1O
informational	They explain what my dental insurance covers.	54.7 (7.3)	Me ayudan con el seguro dental (aseguranza).	77.18 (6.0)	Pay2F	Pay2P	Pay2O
Instrumental	They help me pay for dental care (for example, pay for me, offer me a payment plan, lend me the money).	76.7 (7.7)	Me ayudan a pagar por el dentista (por ejemplo, pagan por mí, me dan opciones de pago, me hacen un préstamo).	99.5 (4.0)	Pay3F	Pay3P	Pay3O
	**WORRY = Help needed to overcome dental worries**		**Miedo dental/preocupación**				
	Do you have any dental fears or worries?	80.3 (4.1)	¿Tiene algún miedo o preocupación dental?	63.98 (7.0/8.0)			
emotional	They listen to any worries about going to the dentist.	69.7 (6.0)	Escuchan cualquier preocupación relacionada con ir al dentista.	45.56 (Cursos Selectivos (difícil))	Worry1F	Worry1P	Worry1O
emotional	They ease my worries about dental treatments.	66.7 (5.6)	Calman mis preocupaciones sobre los tratamientos dentales.	40.67 (sos Selectivos (difícil))	Worry2F	Worry2P	Worry2O
