# Supplementary material for: Development of new bilingual oral health behavior social support (OHBSS) scales in English and Spanish
Source: PLoS One. 2025 Mar 11;20(3):e0317133. doi: 10.1371/journal.pone.0317133 (PMC11896079; doi:10.1371/journal.pone.0317133)
Supplement: S7 Table — (PDF) [file pone.0317133.s007.pdf]

S7 Table. Final OHBSS item descriptives

	Mean (SD)	Median (Mode)	Skew (Kurt)	Mean (SD)	Median (Mode)	Skew (Kurt)	Mean (SD)	Median (Mode)	Skew (Kurt)
	Full sample (N = 502)			English (n = 303)			Spanish (n = 199)		
**BF**									
brush_family_01	1.9 (1.64)	2 (0)	0.09 (-1.62)	1.79 (1.61)	2 (0)	0.18 (-1.57)	2.07 (1.67)	2 (4)	-0.06 (-1.66)
brush_family_02	2.60 (1.56)	3 (4)	-0.65 (-1.14)	2.48 (1.58)	3 (4)	-0.53 (-1.27)	2.77 (1.52)	3 (4)	-0.85 (-0.85)
brush_family_03	1.84 (1.57)	2 (0)	0.14 (-1.5)	1.66 (1.58)	1 (0)	0.32 (-1.45)	2.11 (1.51)	2 (4)	-0.13 (-1.4)
brush_family_04	2.57 (1.45)	3 (4)	-0.56 (-1.06)	2.42 (1.49)	3 (4)	-0.40 (-1.23)	2.81 (1.36)	3 (4)	-0.81 (-0.62)
brush_family_05	2.70 (1.46)	3 (4)	-0.76 (-0.82)	2.54 (1.50)	3 (4)	-0.57 (-1.09)	2.94 (1.37)	4 (4)	-1.10 (-0.11)
brush_family_06	2.09 (1.56)	2 (4)	-0.09 (-1.49)	1.77 (1.53)	2 (0)	0.24 (-1.4)	2.58 (1.48)	3 (4)	-0.63 (-1.02)
brush_family_07	2.33 (1.53)	2.5 (4)	-0.35 (-1.32)	2.19 (1.53)	2 (4)	-0.21 (-1.39)	2.55 (1.49)	3 (4)	-0.57 (-1.11)
brush_family_08	2.19 (1.56)	2 (4)	-0.23 (-1.46)	2.03 (1.61)	2 (0)	-0.05 (-1.57)	2.42 (1.46)	3 (4)	-0.50 (-1.11)
brush_family_09	1.71 (1.54)	2 (0)	0.28 (-1.41)	1.55 (1.55)	1 (0)	0.46 (-1.31)	1.96 (1.51)	2 (0)	0.02 (-1.42)
brush_family_10	2.08 (1.59)	2 (4)	-0.11 (-1.53)	1.97 (1.59)	2 (0)	-0.01 (-1.54)	2.24 (1.57)	3 (4)	-0.27 (-1.47)
brush_family_11	2.59 (1.5)	3 (4)	-0.62 (-1.09)	2.49 (1.51)	3 (4)	-0.51 (-1.21)	2.75 (1.48)	3 (4)	-0.82 (-0.8)
brush_family_12	2.04 (1.61)	2 (0)	-0.08 (-1.57)	1.87 (1.63)	2 (0)	0.12 (-1.6)	2.31 (1.54)	3 (4)	-0.39 (-1.32)
**BP**									
brush_provider_01	2.52 (1.41)	3 (4)	-0.50 (-1.07)	2.53 (1.41)	3 (4)	-0.53 (-1.03)	2.51 (1.42)	3 (4)	-0.46 (-1.12)
brush_provider_02	2.50 (1.51)	3 (4)	-0.51 (-1.21)	2.47 (1.54)	3 (4)	-0.5 (-1.23)	2.55 (1.47)	3 (4)	-0.51 (-1.19)
brush_provider_03	2.74 (1.39)	3 (4)	-0.75 (-0.76)	2.61 (1.46)	3 (4)	-0.61 (-1.05)	2.93 (1.26)	3 (4)	-0.98 (-0.15)
brush_provider_04	2.38 (1.56)	3 (4)	-0.43 (-1.34)	2.22 (1.61)	3 (4)	-0.26 (-1.52)	2.64 (1.45)	3 (4)	-0.69 (-0.89)
brush_provider_05	3.22 (1.25)	4 (4)	-1.5 (0.98)	3.17 (1.31)	4 (4)	-1.43 (0.67)	3.31 (1.15)	4 (4)	-1.60 (1.48)
brush_provider_06	2.84 (1.42)	3 (4)	-0.93 (-0.54)	2.64 (1.49)	3 (4)	-0.69 (-0.97)	3.15 (1.25)	4 (4)	-1.35 (0.65)
brush_provider_07	3.12 (1.25)	4 (4)	-1.28 (0.45)	3.09 (1.28)	4 (4)	-1.25 (0.34)	3.16 (1.21)	4 (4)	-1.34 (0.66)
brush_provider_08	2.99 (1.35)	4 (4)	-1.1 (-0.11)	2.89 (1.41)	4 (4)	-0.99 (-0.42)	3.15 (1.24)	4 (4)	-1.28 (0.48)
brush_provider_09	2.93 (1.36)	4 (4)	-1.04 (-0.2)	2.89 (1.42)	4 (4)	-1.02 (-0.36)	2.99 (1.26)	4 (4)	-1.05 (0.01)
brush_provider_10	2.66 (1.45)	3 (4)	-0.71 (-0.88)	2.52 (1.49)	3 (4)	-0.56 (-1.12)	2.86 (1.37)	3 (4)	-0.97 (-0.34)
brush_provider_11	3.16 (1.26)	4 (4)	-1.43 (0.87)	3.12 (1.28)	4 (4)	-1.33 (0.57)	3.22 (1.23)	4 (4)	-1.61 (1.48)
brush_provider_12	2.53 (1.43)	3 (4)	-0.60 (-0.97)	2.43 (1.5)	3 (4)	-0.50 (-1.2)	2.68 (1.32)	3 (4)	-0.73 (-0.56)
**BO**									
brush_Others_01	0.80 (1.23)	0 (0)	1.44 (0.86)	0.78 (1.23)	0 (0)	1.43 (0.8)	0.82 (1.24)	0 (0)	1.46 (1)
brush_Others_02	0.82 (1.27)	0 (0)	1.39 (0.66)	0.85 (1.31)	0 (0)	1.33 (0.41)	0.78 (1.20)	0 (0)	1.50 (1.14)
brush_Others_03	0.76 (1.23)	0 (0)	1.52 (1.08)	0.74 (1.24)	0 (0)	1.51 (0.99)	0.78 (1.21)	0 (0)	1.54 (1.29)
brush_Others_04	0.82 (1.25)	0 (0)	1.41 (0.77)	0.79 (1.26)	0 (0)	1.43 (0.76)	0.86 (1.24)	0 (0)	1.39 (0.84)
brush_Others_05	1.26 (1.45)	1 (0)	0.8 (-0.78)	1.25 (1.47)	1 (0)	0.80 (-0.8)	1.28 (1.43)	1 (0)	0.79 (-0.72)
brush_Others_06	0.96 (1.35)	0 (0)	1.15 (-0.03)	0.84 (1.32)	0 (0)	1.38 (0.54)	1.15 (1.37)	1 (0)	0.86 (-0.56)
brush_Others_07	0.98 (1.35)	0 (0)	1.16 (-0.03)	0.95 (1.38)	0 (0)	1.22 (0.1)	1.02 (1.32)	0 (0)	1.05 (-0.19)
brush_Others_08	0.97 (1.31)	0 (0)	1.14 (0)	0.95 (1.37)	0 (0)	1.18 (-0.03)	1.01 (1.23)	1 (0)	1.06 (0.06)
brush_Others_09	0.75 (1.25)	0 (0)	1.55 (1.12)	0.74 (1.3)	0 (0)	1.60 (1.16)	0.76 (1.18)	0 (0)	1.46 (1.03)
brush_Others_10	0.88 (1.33)	0 (0)	1.32 (0.35)	0.88 (1.38)	0 (0)	1.32 (0.26)	0.88 (1.27)	0 (0)	1.30 (0.51)
brush_Others_11	1.10 (1.43)	0 (0)	0.98 (-0.49)	1.10 (1.48)	0 (0)	0.99 (-0.58)	1.10 (1.35)	0 (0)	0.95 (-0.35)
brush_Others_12	0.96 (1.37)	0 (0)	1.14 (-0.12)	0.97 (1.41)	0 (0)	1.12 (-0.26)	0.95 (1.3)	0 (0)	1.17 (0.14)

	Mean (SD)	Median (Mode)	Skew (Kurt)	Mean (SD)	Median (Mode)	Skew (Kurt)	Mean (SD)	Median (Mode)	Skew (Kurt)
	**Full sample (N = 502)**			**English (n = 303)**			**Spanish (n = 199)**		
**FF**									
floss_family_01	1.69 (1.50)	2 (0)	0.24 (-1.34)	1.54 (1.48)	1 (0)	0.4 (-1.24)	1.92 (1.49)	2 (0)	0.01 (-1.35)
floss_family_02	1.99 (1.52)	2 (0)	-0.03 (-1.44)	1.83 (1.50)	2 (0)	0.11 (-1.41)	2.23 (1.53)	2 (4)	-0.25 (-1.38)
floss_family_03	1.61 (1.51)	1 (0)	0.33 (-1.37)	1.44 (1.49)	1 (0)	0.54 (-1.17)	1.88 (1.52)	2 (0)	0.03 (-1.44)
floss_family_04	2.57 (1.45)	3 (4)	-0.56 (-1.06)	2.42 (1.49)	3 (4)	-0.4 (-1.23)	2.81 (1.36)	3 (4)	-0.81 (-0.62)
floss_family_05	2.03 (1.53)	2 (0)	-0.10 (-1.44)	1.9 (1.52)	2 (0)	0.04 (-1.44)	2.22 (1.52)	2 (4)	-0.31 (-1.35)
floss_family_06	1.66 (1.53)	2 (0)	0.28 (-1.41)	1.56 (1.53)	1 (0)	0.38 (-1.35)	1.8 (1.51)	2 (0)	0.14 (-1.44)
floss_family_07	1.80 (1.52)	2 (0)	0.15 (-1.44)	1.72 (1.55)	2 (0)	0.24 (-1.45)	1.93 (1.48)	2 (0)	0 (-1.39)
floss_family_08	1.67 (1.56)	2 (0)	0.29 (-1.43)	1.56 (1.57)	1 (0)	0.41 (-1.39)	1.82 (1.52)	2 (0)	0.14 (-1.43)
floss_family_09	1.52 (1.51)	1 (0)	0.42 (-1.3)	1.45 (1.51)	1 (0)	0.51 (-1.25)	1.62 (1.49)	2 (0)	0.29 (-1.33)
floss_family_10	1.65 (1.55)	1 (0)	0.31 (-1.43)	1.54 (1.56)	1 (0)	0.43 (-1.36)	1.8 (1.54)	2 (0)	0.12 (-1.47)
floss_family_11	1.94 (1.55)	2 (0)	0.02 (-1.49)	1.88 (1.54)	2 (0)	0.08 (-1.46)	2.04 (1.56)	2 (0)	-0.08 (-1.53)
floss_family_12	1.60 (1.56)	1 (0)	0.33 (-1.44)	1.5 (1.56)	1 (0)	0.45 (-1.37)	1.75 (1.54)	2 (0)	0.15 (-1.49)
**FP**									
floss_provider_01	2.86 (1.38)	3 (4)	-0.93 (-0.44)	2.79 (1.4)	3 (4)	-0.85 (-0.56)	2.96 (1.36)	4 (4)	-1.07 (-0.19)
floss_provider_02	2.42 (1.55)	3 (4)	-0.48 (-1.28)	2.38 (1.59)	3 (4)	-0.43 (-1.38)	2.48 (1.5)	3 (4)	-0.55 (-1.12)
floss_provider_03	2.80 (1.4)	3 (4)	-0.83 (-0.65)	2.71 (1.42)	3 (4)	-0.72 (-0.82)	2.93 (1.37)	4 (4)	-1.03 (-0.28)
floss_provider_04	2.38 (1.56)	3 (4)	-0.43 (-1.34)	2.22 (1.61)	3 (4)	-0.26 (-1.52)	2.64 (1.45)	3 (4)	-0.69 (-0.89)
floss_provider_05	3.14 (1.27)	4 (4)	-1.37 (0.66)	3.14 (1.27)	4 (4)	-1.36 (0.66)	3.15 (1.28)	4 (4)	-1.39 (0.71)
floss_provider_06	2.69 (1.47)	3 (4)	-0.72 (-0.92)	2.62 (1.5)	3 (4)	-0.65 (-1.03)	2.79 (1.43)	3 (4)	-0.83 (-0.73)
floss_provider_07	2.94 (1.37)	4 (4)	-1.03 (-0.27)	2.94 (1.38)	4 (4)	-1.04 (-0.25)	2.93 (1.36)	4 (4)	-1.02 (-0.28)
floss_provider_08	2.81 (1.44)	3 (4)	-0.88 (-0.65)	2.82 (1.47)	4 (4)	-0.89 (-0.66)	2.81 (1.39)	3 (4)	-0.85 (-0.63)
floss_provider_09	2.73 (1.48)	3 (4)	-0.77 (-0.89)	2.77 (1.46)	3 (4)	-0.8 (-0.79)	2.68 (1.51)	3 (4)	-0.72 (-1.02)
floss_provider_10	2.50 (1.5)	3 (4)	-0.54 (-1.14)	2.44 (1.5)	3 (4)	-0.48 (-1.18)	2.59 (1.5)	3 (4)	-0.64 (-1.06)
floss_provider_11	3.00 (1.35)	4 (4)	-1.12 (-0.07)	3.01 (1.34)	4 (4)	-1.14 (0.03)	2.98 (1.38)	4 (4)	-1.1 (-0.18)
floss_provider_12	2.28 (1.55)	2.5 (4)	-0.31 (-1.4)	2.17 (1.57)	2 (4)	-0.19 (-1.46)	2.45 (1.52)	3 (4)	-0.49 (-1.23)
**FO**									
floss_Others_01	0.71 (1.15)	0 (0)	1.56 (1.35)	0.70 (1.18)	0 (0)	1.61 (1.42)	0.72 (1.11)	0 (0)	1.49 (1.24)
floss_Others_02	0.74 (1.19)	0 (0)	1.51 (1.12)	0.72 (1.2)	0 (0)	1.57 (1.32)	0.76 (1.19)	0 (0)	1.42 (0.87)
floss_Others_03	0.65 (1.14)	0 (0)	1.71 (1.81)	0.65 (1.18)	0 (0)	1.75 (1.85)	0.64 (1.08)	0 (0)	1.64 (1.70)
floss_Others_04	0.82 (1.25)	0 (0)	1.41 (0.77)	0.79 (1.26)	0 (0)	1.43 (0.76)	0.86 (1.24)	0 (0)	1.39 (0.84)
floss_Others_05	0.84 (1.27)	0 (0)	1.36 (0.58)	0.87 (1.32)	0 (0)	1.34 (0.45)	0.80 (1.20)	0 (0)	1.38 (0.79)
floss_Others_06	0.76 (1.24)	0 (0)	1.52 (1.02)	0.79 (1.31)	0 (0)	1.50 (0.85)	0.71 (1.13)	0 (0)	1.51 (1.15)
floss_Others_07	0.75 (1.26)	0 (0)	1.57 (1.13)	0.77 (1.32)	0 (0)	1.54 (0.92)	0.71 (1.17)	0 (0)	1.59 (1.43)
floss_Others_08	0.75 (1.25)	0 (0)	1.53 (1.07)	0.79 (1.32)	0 (0)	1.53 (0.93)	0.69 (1.12)	0 (0)	1.47 (0.99)
floss_Others_09	0.70 (1.19)	0 (0)	1.65 (1.55)	0.71 (1.24)	0 (0)	1.67 (1.53)	0.68 (1.11)	0 (0)	1.59 (1.47)
floss_Others_10	0.71 (1.23)	0 (0)	1.63 (1.38)	0.72 (1.28)	0 (0)	1.66 (1.41)	0.71 (1.17)	0 (0)	1.55 (1.25)
floss_Others_11	0.80 (1.25)	0 (0)	1.48 (0.98)	0.84 (1.31)	0 (0)	1.42 (0.72)	0.74 (1.16)	0 (0)	1.57 (1.46)
floss_Others_12	0.71 (1.24)	0 (0)	1.61 (1.28)	0.70 (1.27)	0 (0)	1.65 (1.35)	0.72 (1.2)	0 (0)	1.55 (1.20)

	Mean (SD)	Median (Mode)	Skew (Kurt)	Mean (SD)	Median (Mode)	Skew (Kurt)	Mean (SD)	Median (Mode)	Skew (Kurt)
	**Full sample (N = 502)**			**English (n = 303)**			**Spanish (n = 199)**		
**DF**									
dentcare_fam_01	2.22 (1.54)	2 (4)	-0.22 (-1.42)	2.11 (1.59)	2 (4)	-0.1 (-1.52)	2.39 (1.46)	3 (4)	-0.41 (-1.20)
dentcare_fam_02	2.15 (1.59)	2 (4)	-0.18 (-1.54)	1.93 (1.62)	2 (0)	0.06 (-1.60)	2.48 (1.49)	3 (4)	-0.56 (-1.13)
dentcare_fam_03	2.11 (1.58)	2 (4)	-0.11 (-1.53)	1.98 (1.62)	2 (0)	0.01 (-1.59)	2.30 (1.51)	2 (4)	-0.28 (-1.39)
dentcare_fam_04	1.40 (1.50)	1 (0)	0.54 (-1.20)	1.26 (1.49)	0 (0)	0.72 (-1.00)	1.61 (1.48)	2 (0)	0.29 (-1.35)
dentcare_fam_05	1.43 (1.49)	1 (0)	0.52 (-1.20)	1.28 (1.49)	1 (0)	0.72 (-0.99)	1.66 (1.47)	2 (0)	0.26 (-1.33)
dentcare_fam_06	2.57 (1.47)	3 (4)	-0.6 (-1.06)	2.47 (1.50)	3 (4)	-0.50 (-1.20)	2.74 (1.42)	3 (4)	-0.76 (-0.79)
dentcare_fam_07	1.87 (1.56)	2 (0)	0.08 (-1.50)	1.68 (1.58)	2 (0)	0.29 (-1.47)	2.17 (1.47)	2 (4)	-0.22 (-1.33)
dentcare_fam_08	2.48 (1.42)	3 (4)	-0.49 (-1.07)	2.47 (1.43)	3 (4)	-0.44 (-1.12)	2.49 (1.42)	3 (4)	-0.56 (-1.00)
dentcare_fam_09	2.38 (1.45)	3 (4)	-0.39 (-1.19)	2.35 (1.46)	2 (4)	-0.32 (-1.25)	2.44 (1.42)	3 (4)	-0.5 (-1.06)
dentcare_fam_10	2.21 (1.55)	2 (4)	-0.21 (-1.46)	2.13 (1.58)	2 (4)	-0.12 (-1.51)	2.32 (1.51)	3 (4)	-0.35 (-1.35)
dentcare_fam_11	1.89 (1.49)	2 (0)	0.03 (-1.40)	1.79 (1.52)	2 (0)	0.20 (-1.39)	2.05 (1.43)	2 (3)	-0.22 (-1.28)
dentcare_fam_12	1.93 (1.53)	2 (0)	0.04 (-1.45)	1.86 (1.55)	2 (0)	0.10 (-1.47)	2.03 (1.5)	2 (0)	-0.06 (-1.41)
dentcare_fam_13	2.81 (1.38)	3 (4)	-0.86 (-0.55)	2.84 (1.38)	3 (4)	-0.87 (-0.55)	2.75 (1.38)	3 (4)	-0.84 (-0.55)
dentcare_fam_14	1.89 (1.57)	2 (0)	0.06 (-1.50)	1.88 (1.58)	2 (0)	0.07 (-1.51)	1.89 (1.55)	2 (0)	0.04 (-1.49)
**DP**									
dentcare_prov_01	2.59 (1.42)	3 (4)	-0.59 (-0.98)	2.41 (1.49)	3 (4)	-0.40 (-1.25)	2.87 (1.27)	3 (4)	-0.87 (-0.33)
dentcare_prov_02	2.46 (1.49)	3 (4)	-0.51 (-1.16)	2.27 (1.54)	3 (4)	-0.33 (-1.39)	2.75 (1.37)	3 (4)	-0.80 (-0.55)
dentcare_prov_03	2.92 (1.38)	4 (4)	-1.03 (-0.25)	2.81 (1.43)	3 (4)	-0.90 (-0.56)	3.08 (1.29)	4 (4)	-1.26 (0.42)
dentcare_prov_04	3.02 (1.31)	4 (4)	-1.17 (0.14)	2.91 (1.37)	4 (4)	-1.00 (-0.30)	3.20 (1.19)	4 (4)	-1.46 (1.14)
dentcare_prov_05	3.11 (1.21)	4 (4)	-1.29 (0.68)	3.07 (1.26)	4 (4)	-1.26 (0.50)	3.18 (1.13)	4 (4)	-1.32 (0.93)
dentcare_prov_06	3.14 (1.28)	4 (4)	-1.33 (0.51)	3.07 (1.31)	4 (4)	-1.23 (0.21)	3.25 (1.22)	4 (4)	-1.51 (1.13)
dentcare_prov_07	3.18 (1.25)	4 (4)	-1.42 (0.81)	3.14 (1.25)	4 (4)	-1.35 (0.65)	3.24 (1.24)	4 (4)	-1.54 (1.15)
dentcare_prov_08	3.11 (1.2)	4 (4)	-1.27 (0.60)	3.07 (1.22)	4 (4)	-1.19 (0.34)	3.18 (1.17)	4 (4)	-1.42 (1.13)
dentcare_prov_09	3.08 (1.28)	4 (4)	-1.24 (0.33)	2.99 (1.33)	4 (4)	-1.10 (-0.08)	3.22 (1.17)	4 (4)	-1.48 (1.19)
dentcare_prov_10	2.47 (1.57)	3 (4)	-0.49 (-1.31)	2.37 (1.62)	3 (4)	-0.39 (-1.45)	2.63 (1.47)	3 (4)	-0.63 (-1.03)
dentcare_prov_11	2.79 (1.38)	3 (4)	-0.81 (-0.59)	2.72 (1.39)	3 (4)	-0.75 (-0.70)	2.89 (1.34)	3 (4)	-0.92 (-0.39)
dentcare_prov_12	2.36 (1.52)	3 (4)	-0.38 (-1.31)	2.18 (1.55)	2 (4)	-0.18 (-1.46)	2.64 (1.44)	3 (4)	-0.71 (-0.83)
dentcare_prov_13	3.03 (1.28)	4 (4)	-1.18 (0.27)	3.00 (1.31)	4 (4)	-1.11 (0.03)	3.09 (1.23)	4 (4)	-1.30 (0.74)
dentcare_prov_14	2.6 (1.47)	3 (4)	-0.63 (-1.00)	2.38 (1.51)	3 (4)	-0.37 (-1.29)	2.95 (1.33)	4 (4)	-1.08 (-0.03)
**DO**									
dentcare_others_01	0.88 (1.26)	0 (0)	1.26 (0.38)	0.79 (1.27)	0 (0)	1.49 (0.92)	1.03 (1.24)	1 (0)	0.96 (-0.17)
dentcare_others_02	0.98 (1.30)	0 (0)	1.05 (-0.16)	0.87 (1.31)	0 (0)	1.27 (0.26)	1.16 (1.27)	1 (0)	0.78 (-0.54)
dentcare_others_03	0.78 (1.29)	0 (0)	1.50 (0.90)	0.79 (1.34)	0 (0)	1.51 (0.81)	0.77 (1.2)	0 (0)	1.46 (1.00)
dentcare_others_04	0.70 (1.18)	0 (0)	1.64 (1.59)	0.67 (1.19)	0 (0)	1.72 (1.85)	0.74 (1.18)	0 (0)	1.53 (1.29)
dentcare_others_05	0.75 (1.24)	0 (0)	1.53 (1.09)	0.72 (1.24)	0 (0)	1.59 (1.26)	0.79 (1.24)	0 (0)	1.45 (0.91)
dentcare_others_06	1.20 (1.45)	0.5 (0)	0.83 (-0.73)	1.10 (1.45)	0 (0)	1.00 (-0.46)	1.35 (1.43)	1 (0)	0.61 (-0.98)
dentcare_others_07	0.96 (1.34)	0 (0)	1.18 (0.07)	0.87 (1.34)	0 (0)	1.38 (0.55)	1.09 (1.33)	0 (0)	0.91 (-0.45)
dentcare_others_08	1.20 (1.39)	1 (0)	0.81 (-0.67)	1.21 (1.41)	1 (0)	0.83 (-0.65)	1.20 (1.37)	1 (0)	0.78 (-0.69)

dentcare_others_09	1.05 (1.35)	0 (0)	0.98 (-0.38)	1.02 (1.39)	0 (0)	1.08 (-0.26)	1.1 (1.28)	1 (0)	0.81 (-0.57)
dentcare_others_10	1.10 (1.42)	0 (0)	0.96 (-0.54)	1.05 (1.45)	0 (0)	1.05 (-0.39)	1.18 (1.39)	1 (0)	0.82 (-0.73)
dentcare_others_11	0.88 (1.25)	0 (0)	1.30 (0.54)	0.88 (1.30)	0 (0)	1.34 (0.54)	0.87 (1.18)	0 (0)	1.21 (0.49)
dentcare_others_12	0.96 (1.31)	0 (0)	1.11 (-0.07)	0.94 (1.32)	0 (0)	1.15 (-0.02)	1.00 (1.28)	0 (0)	1.04 (-0.11)
dentcare_others_13	1.39 (1.50)	1 (0)	0.61 (-1.10)	1.43 (1.55)	1 (0)	0.55 (-1.25)	1.33 (1.42)	1 (0)	0.70 (-0.84)
dentcare_others_14	0.85 (1.28)	0 (0)	1.33 (0.51)	0.87 (1.31)	0 (0)	1.34 (0.48)	0.82 (1.22)	0 (0)	1.30 (0.52)
**OPTIONAL DENTAL CARE***									
	Mean (SD)	Median (Mode)	Skew (Kurt)	Mean (SD)	Median (Mode)	Skew (Kurt)	Mean (SD)	Median (Mode)	Skew (Kurt)
	Full sample			English			Spanish		
**Translate**	(n=112)			(n=1)			(n=111)		
dentcare_fam_translate	2.45 (1.48)	3 (4)	-0.47 (-1.13)	4	4 (4)	-	2.43 (1.48)	3 (4)	-0.46 (-1.14)
dentcare_prov_translate	3.35 (0.99)	4 (4)	-1.65 (2.31)	1	1 (1)	-	3.34 (1.00)	4 (4)	-1.64 (2.26)
dentcare_others_translate	1.18 (1.34)	1 (0)	0.85 (-0.43)	1	1 (1)	-	1.18 (1.34)	1 (0)	0.85 (-0.43)
**Transportation**	(n=69)			(n=29)			(n=40)		
dentcare_fam_transport	2.61 (1.36)	3 (4)	-0.68 (-0.64)	2.69 (1.54)	3 (4)	-0.83 (-0.84)	2.55 (1.24)	3 (2)	-0.59 (-0.32)
dentcare_prov_transport	1.25 (1.58)	0 (0)	0.83 (-0.96)	0.86 (1.51)	0 (0)	1.47 (0.45)	1.53 (1.58)	1 (0)	1.47 (0.45)
dentcare_others_transport	1.22 (1.33)	1 (0)	0.71 (-0.62)	1.00 (1.34)	0 (0)	1.06 (-0.16)	1.38 (1.31)	1.5 (0)	0.53 (-0.64)
**Pay**	(n=242)			(n=137)			(n=105)		
dentcare_fam_Pay1	1.44 (1.49)	1 (0)	0.56 (-1.13)	1.31 (1.53)	1 (0)	0.77 (-0.92)	1.61 (1.43)	2 (0)	0.30 (-1.25)
dentcare_prov_Pay1	2.06 (1.51)	2 (4)	-0.04 (-1.41)	1.80 (1.51)	2 (0)	0.26 (-1.34)	2.40 (1.45)	3 (4)	-0.44 (-1.10)
dentcare_others_Pay1	0.67 (1.08)	0 (0)	1.59 (1.66)	0.58 (1.01)	0 (0)	1.82 (2.67)	0.79 (1.17)	0 (0)	1.36 (0.83)
dentcare_fam_Pay2	1.12 (1.42)	0 (0)	0.98 (-0.45)	0.91 (1.35)	0 (0)	1.34 (0.42)	1.39 (1.48)	1 (0)	0.63 (-1.01)
dentcare_prov_Pay2	1.98 (1.50)	2 (4)	0.06 (-1.38)	2.07 (1.51)	2 (4)	0.00 (-1.42)	1.86 (1.48)	2 (4)	0.12 (-1.32)
dentcare_others_Pay2	0.37 (0.83)	0 (0)	2.53 (6.11)	0.29 (0.70)	0 (0)	2.95 (9.53)	0.47 (0.96)	0 (0)	2.11 (3.67)
dentcare_fam_Pay3	1.58 (1.65)	1 (0)	0.66 (-0.96)	1.05 (1.33)	0 (0)	0.97 (-0.28)	1.61 (1.55)	2 (0)	0.31 (-1.40)
dentcare_prov_Pay3	1.31 (1.46)	0 (0)	0.83 (-0.96)	0.86 (1.51)	0 (0)	1.47 (0.45)	1.53 (1.58)	1 (0)	1.47 (0.45)
dentcare_others_Pay3	0.35 (0.91)	0 (0)	2.73 (6.95)	0.26 (0.77)	0 (0)	3.59 (13.23)	0.50 (1.05)	0 (0)	2.11 (3.52)
**Worries**	(n=172)			(n=99)			(n=73)		
dentcare_fam_worry1	2.49 (1.45)	3 (4)	-0.42 (-1.19)	2.47 (1.51)	3 (4)	-0.44 (-1.29)	2.51 (1.37)	2 (4)	-0.39 (-1.05)
dentcare_prov_worry1	2.09 (1.52)	2 (4)	-0.09 (-1.42)	1.85 (1.55)	2 (0)	0.17 (-1.46)	2.42 (1.41)	3 (4)	-0.43 (-1.04)
dentcare_others_worry1	1.29 (1.40)	1 (0)	0.70 (-0.81)	1.37 (1.50)	1 (0)	0.66 (-0.16)	1.18 (1.25)	1 (0)	0.66 (-0.66)
dentcare_fam_worry2	2.16 (1.55)	2 (4)	-0.16 (-1.47)	2.12 (1.58)	2 (4)	-0.12 (-1.51)	2.22 (1.51)	2 (4)	-0.21 (-1.44)
dentcare_prov_worry2	2.13 (1.51)	2 (4)	-0.14 (-1.41)	1.90 (1.52)	2 (0)	0.08 (-1.42)	2.45 (1.45)	3 (4)	-0.45 (-1.15)
dentcare_others_worry2	1.10 (1.33)	0 (0)	0.81 (-0.67)	1.15 (1.39)	0 (0)	0.81 (-0.67)	1.15 (1.39)	0 (0)	0.77 (-0.71)
